# Heterostructured Core–Shell Ni–Co@Fe–Co Nanoboxes of Prussian Blue Analogues for Efficient Electrocatalytic Hydrogen Evolution from Alkaline Seawater

**DOI:** 10.1021/acscatal.2c05433

**Published:** 2023-01-09

**Authors:** Hao Zhang, Jiefeng Diao, Mengzheng Ouyang, Hossein Yadegari, Mingxuan Mao, Mengnan Wang, Graeme Henkelman, Fang Xie, D. Jason Riley

**Affiliations:** † Department of Materials and London Center for Nanotechnology, Imperial College London, London SW7 2AZ, U.K.; ‡ Department of Chemistry and the Oden Institute for Computational Engineering and Sciences, The University of Texas at Austin, Austin, Texas 78712 United States; § Department of Earth Science and Engineering, Imperial College London, London SW7 2AZ U.K.; ∥ Department of Electrical and Electronic Engineering, Imperial College London, London SW7 2AZ U.K.

**Keywords:** Prussian blue analogue, heterostructure, trimetallic nanobox, hydrogen evolution, seawater splitting

## Abstract

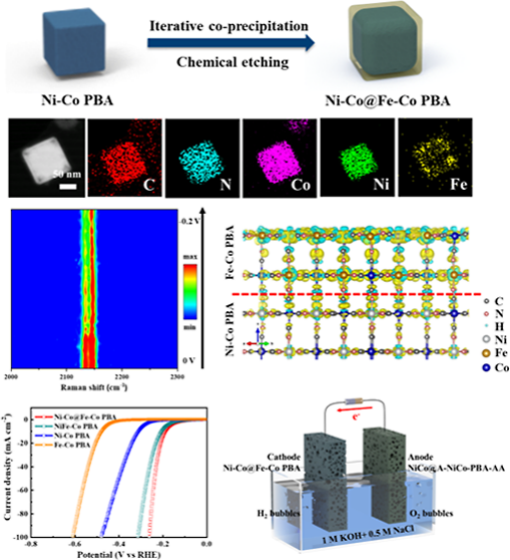

The rational construction of efficient and low-cost electrocatalysts for the hydrogen evolution reaction (HER) is critical to seawater electrolysis. Herein, trimetallic heterostructured core–shell nanoboxes based on Prussian blue analogues (Ni–Co@Fe–Co PBA) were synthesized using an iterative coprecipitation strategy. The same coprecipitation procedure was used for the preparation of the PBA core and shell, with the synthesis of the shell involving chemical etching during the introduction of ferrous ions. Due to its unique structure and composition, the optimized trimetallic Ni–Co@Fe–Co PBA possesses more active interfacial sites and a high specific surface area. As a result, the developed Ni–Co@Fe–Co PBA electrocatalyst exhibits remarkable electrocatalytic HER performance with small overpotentials of 43 and 183 mV to drive a current density of 10 mA cm^–2^ in alkaline freshwater and simulated seawater, respectively. Operando Raman spectroscopy demonstrates the evolution of Co^2+^ from Co^3+^ in the catalyst during HER. Density functional theory simulations reveal that the H*–N adsorption sites lower the barrier energy of the rate-limiting step, and the introduced Fe species improve the electron mobility of Ni–Co@Fe–Co PBA. The charge transfer at the core–shell interface leads to the generation of H* intermediates, thereby enhancing the HER activity. By pairing this HER catalyst (Ni–Co@Fe–Co PBA) with another core–shell PBA OER catalyst (NiCo@A-NiCo-PBA-AA) reported by our group, the fabricated two-electrode electrolyzer was found to achieve high output current densities of 44 and 30 mA cm^–2^ at a low voltage of 1.6 V in alkaline freshwater and simulated seawater, respectively, exhibiting remarkable durability over a 100 h test.

## 1 Introduction

To alleviate the energy crisis and air pollution caused by using fossil fuels, the development of clean and renewable energy has attracted widespread attention.^1−3^ Hydrogen is a potential clean energy because of its high energy density, zero-carbon emission, and sustainability, and the electrocatalytic hydrogen evolution reaction (HER) is highly promising for the production of hydrogen with high purity.^4,5^ Seawater is the most abundant natural electrolyte on earth, and seawater electrolysis is a “hits two birds with one stone” technology that can be used for both hydrogen production and seawater desalination, which is ideal for low-cost and large-scale production of hydrogen.^6−8^ However, the design of HER electrocatalysts for operation in seawater is challenging due to ion poisoning and high corrosivity.^9,10^

Preparation of trimetallic compounds is an effective strategy to improve electrocatalytic performance.^11,12^ The extent of structural distortion and defects can be generated due to the interaction of the different ionic radii and electronic properties of the constituent metallic ions, improving the conductivity and enlarging the electrochemically active surface area (ECSA).^13^ The interaction between different metal elements also effectively tunes the 3d electronic structure, thereby allowing optimization of the dissociation energy of water and the adsorption energy of intermediates to promote HER activity.^14^ Furthermore, the combination of trimetal elements may produce a variety of binding sites, which serve as active centers and improve the intrinsic activity of the catalyst.^15^

Prussian blue analogues (PBAs) have been extensively studied as coordination materials for electrocatalytic applications. For example, Zhang and co-workers reported 3D PBA cubes deposited on 2D metal hydroxides/oxides for water splitting under alkaline conditions.^16^ Wu et al. used tannic acid to etch anion- and cation-rich bivacancies (V_CN_ and V_Co_) within CoFe-PBA as an alkaline HER electrocatalyst.^17^ The tunable metal nodes of PBAs meet the requirements for the preparation of multimetallic compounds, and the adjustable open frameworks permit structural designability, providing opportunities for the design of complex structures.^18−20^

Core–shell structured PBAs generally ensure large surface areas and structural stability, which increase the electrode–electrolyte contact area and the number of active sites.^21−23^ This structure also reduces the diffusion distance of charges and ions, resulting in low potential and high activity and stability of electrocatalysts.^24−26^ However, previous core–shell PBAs have been of the homogeneous core and shell, which is not conducive to synergy between components, and core–shell PBAs with both heterogeneous core and shell are rarely reported.

Herein, well-designed trimetallic Ni–Co@Fe–Co PBA nanoboxes with heterostructured core–shell architectures were synthesized through an iterative coprecipitation method. Ni–Co PBA truncated nanoboxes were prepared as the core using potassium hexacyanocobaltate(III) as the organic linker and nickel ions as metal nodes. The same ligands and ferrous ion nodes were subsequently used to synthesize the Fe–Co PBA shell under the same reaction conditions, enclosing the highly active Ni–Co PBA core in the robust Fe–Co PBA shell. The trimetallic components were characterized using X-ray diffraction (XRD), X-ray photoelectron spectroscopy (XPS), and high-angle annular dark-field scanning transmission electron microscopy (HAADF–STEM) with elemental mapping. The HER performance of the material was tested in alkaline freshwater and seawater. Ni–Co@Fe–Co PBA exhibits much lower overpotential and higher durability due to the synergy of the high activity of the Ni–Co PBA core and the high structural stability of the Fe–Co PBA shell compared with the HER performance of the previously reported bifunctional core–shell PBA catalyst (NiCo@A-NiCo-PBA-AA) by our group.^19^ Operando Raman spectroscopy and density functional theory (DFT) calculations were performed to assess the enhanced performance of the material. Finally, the Ni–Co@Fe–Co PBA was used as the cathode and coupled with the NiCo@A-NiCo-PBA-AA anode to form a two-electrode electrolyzer, and the seawater-splitting performance of the device was determined.

## 2 Results and Discussion

### 2.1 Structural Characterization and Chemical Analysis

**Figure 1:**
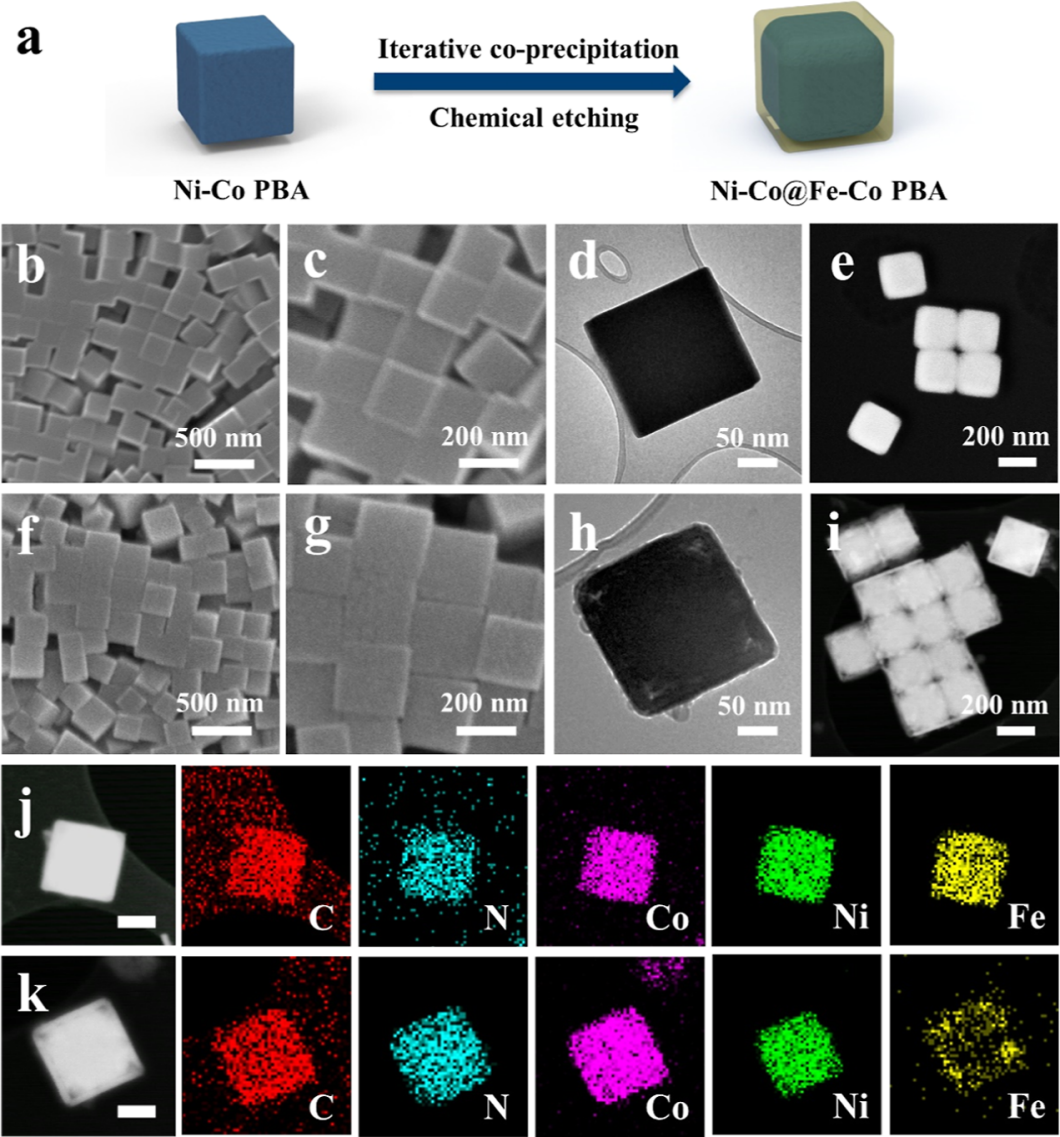
Structural characterizations. (a) Schematic diagram of the synthesis of Ni–Co@Fe–Co PBA. (b,c) FESEM, (d) TEM, and (e) HAADF–STEM images of NiFe–Co PBA. (f,g) FESEM, (h) TEM, and (i) HAADF–STEM images of Ni–Co@Fe–Co PBA. (j) HAADF–STEM and elemental mapping images of NiFe–Co PBA (scale bar: 50 nm). (k) HAADF–STEM and elemental mapping images of Ni–Co@Fe–Co PBA (scale bar: 50 nm).

The approach for the synthesis of heterogeneous Ni–Co@Fe–Co PBA nanoboxes is illustrated in Figure 1a; it involves coprecipitation and chemical etching procedures. Ni–Co PBA (Ni_3_[Co^III^(CN)_6_]_2_) truncated nanoboxes were prepared as the core by dissolving nickel ion nodes, potassium hexacyanocobaltate(III) linkers, and citrate dihydrate capping agents in deionized water and aging for 24 h (eq 1). Ferrous ions were subsequently introduced using the same method to synthesize a Fe–Co PBA (Fe_3_[Co^III^(CN)_6_]_2_) shell (eq 2). The solubility product constant (*K*_sp_) of Fe_3_[Co^III^(CN)_6_]_2_ (*K*_sp_ = 2.4 × 10^–40^) is smaller than that of Ni_3_[Co^III^(CN)_6_]_2_ (*K*_sp_ = 1.6 × 10^–13^),^27,28^ and hence ferrous ions selectively etch the corners of the core Ni–Co PBA (eqs 3 and 4). This resulted in a gradual transformation from a truncated cubic to a spherical core structure. The in situ generated Fe–Co PBA shell wrapped around the spherical core, forming a well-defined core–shell nanobox. The preparation of NiFe–Co PBA nanoboxes, for control experiments, was similar to that of the core Ni–Co PBA; nickel ions and ferrous ions, which serve as metal nodes, were mixed with potassium hexacyanocobaltate(III) and citrate dihydrate, and the resulting solution was aged at room temperature for 24 h (eq 5).

Ni–Co@Fe–Co PBA
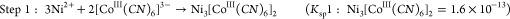
1
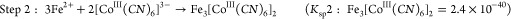
2
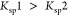

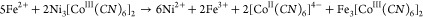
3
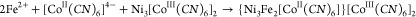
4

NiFe–Co PBA
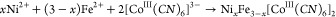
5

Figure S1 shows the field-emission scanning electron microscopy (FESEM) images of Ni–Co PBA nanoboxes, which have relatively smooth surfaces and uniform sizes of ca. 200 nm with truncated vertices compared to those of Fe–Co PBA (Figure S2). Figure 1b,c displays the FESEM images of NiFe–Co PBA. It is observed that NiFe–Co PBA nanoparticles exhibit regular nontruncated cubic structures and highly uniform distribution and are of a size similar to that of Ni–Co PBA. The transmission electron microscopy (TEM) and HAADF–STEM images of NiFe–Co PBA are shown in Figure 1d,e, demonstrating its solid structure. Figure 1f,g shows the FESEM images of Ni–Co@Fe–Co PBA showing a cubic structure similar to that of NiFe–Co PBA, with a relatively rougher surface. The particle size of Ni–Co@Fe–Co PBA is about 230 nm, which is slightly larger than that of NiFe–Co PBA. The core–shell structure of Ni–Co@Fe–Co PBA is clearly observed from the TEM and HAADF–STEM images (Figure 1h,i), in which the core is spherical and the shell is a regular cubic structure. The STEM-mapping images of NiFe–Co PBA and Ni–Co@Fe–Co PBA are displayed in Figure 1j,k, demonstrating that they both are composed of C, N, Co, Ni, and Fe. In NiFe–Co PBA, the five elements are uniformly distributed, while in Ni–Co@Fe–Co PBA, Ni is concentrated in the core, while Fe is mainly distributed in the shell.

**Figure 2:**
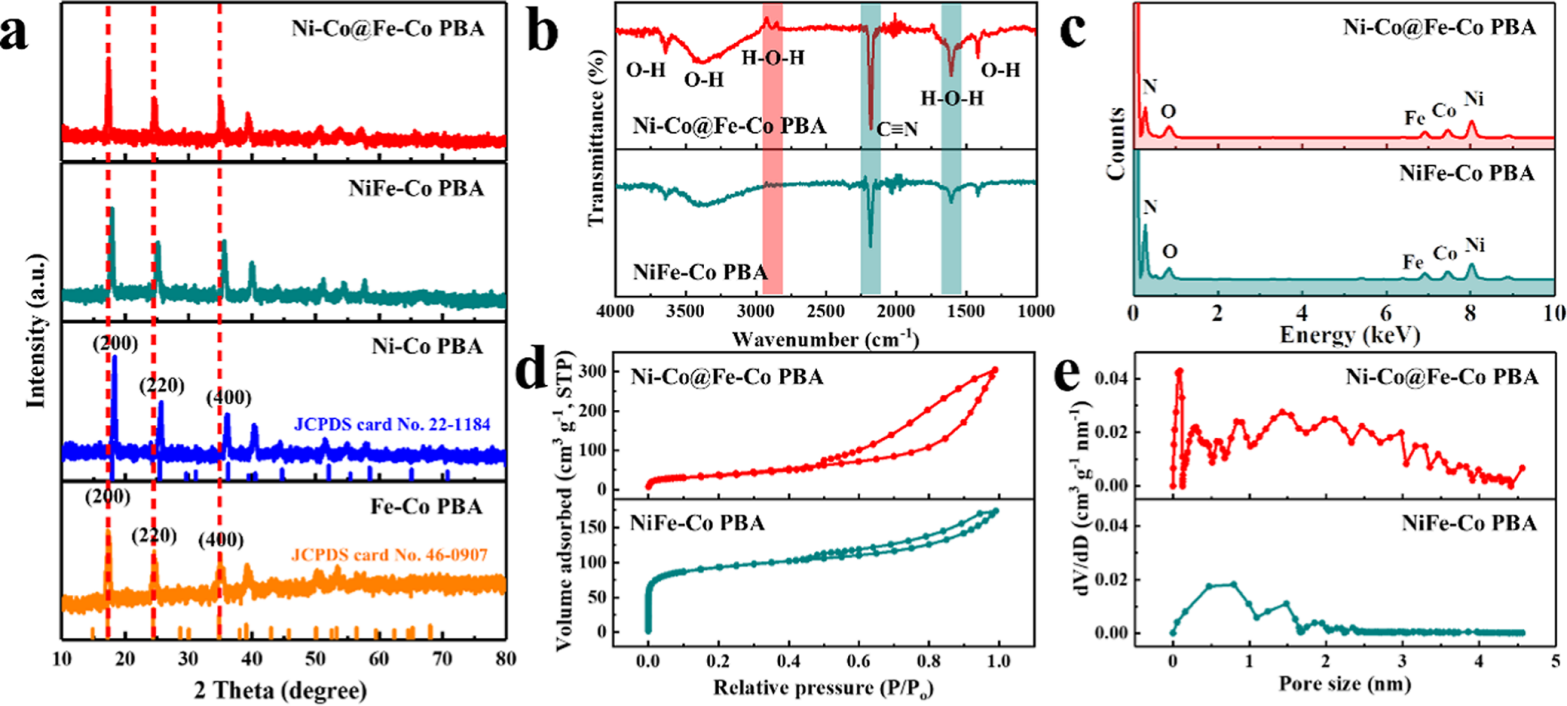
Chemical composition analysis. (a) XRD patterns of Ni–Co@Fe–Co PBA, NiFe–Co PBA, Ni–Co PBA, and Fe–Co PBA. (b) FT-IR spectra of Ni–Co@Fe–Co PBA and NiFe–Co PBA. (c) STEM-EDS spectra of Ni–Co@Fe–Co PBA and NiFe–Co PBA. (d) N_2_ adsorption/desorption isotherms of Ni–Co@Fe–Co PBA and NiFe–Co PBA. © Pore size distribution of Ni–Co@Fe–Co PBA and NiFe–Co PBA.

**Figure 3:**
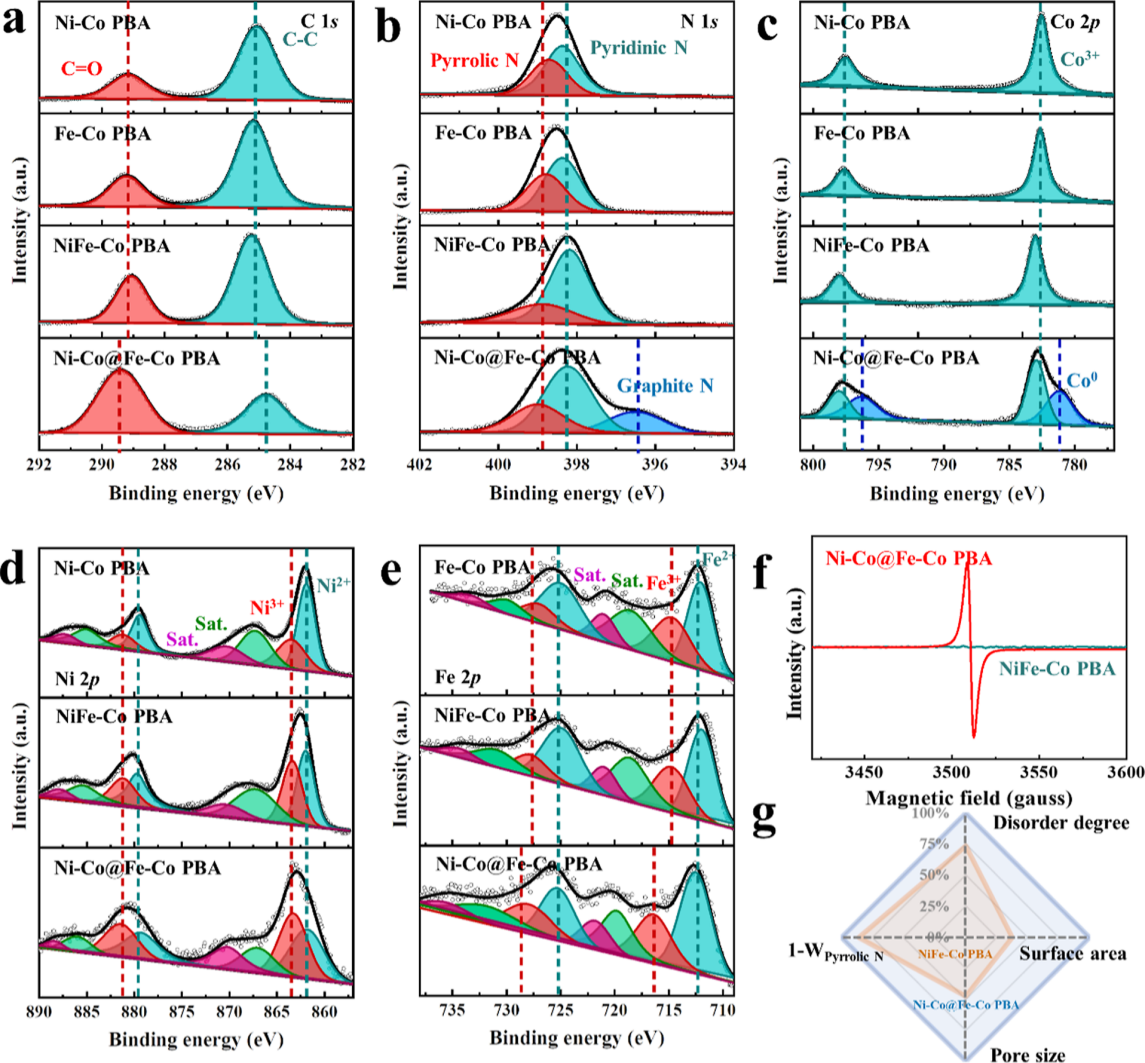
Chemical composition analysis. XPS high-resolution spectra at (a) C 1s edge, (b) N 1s edge, (c) Co 2p edge, (d) Ni 2p edge, and (e) Fe 2p edge of PBA-based catalysts. (f) EPR spectra of Ni–Co@Fe–Co PBA and NiFe–Co PBA. (g) Spider chart of the structural and compositional parameters for the comparison of Ni–Co@Fe–Co PBA (blue) and NiFe–Co PBA (yellow) (the parameters of NiFe–Co PBA were normalized according to Ni–Co@Fe–Co PBA).

Figure 2a shows the XRD patterns of the PBA-based catalysts. The diffraction peaks of the as-obtained Ni–Co PBA and Fe–Co PBA can be well-indexed as Ni_3_[Co(CN)_6_]_2_ (JCPDS card no. 22-1184) and Fe_3_[Co(CN)_6_]_2_ (JCPDS card no. 46-0907), respectively, confirming their high purity and good crystallinity. The XRD peaks of NiFe–Co PBA are between those of Ni–Co PBA and Fe–Co PBA, implying that their lattice spacings are between Ni–Co PBA and Fe–Co PBA. The diffraction peaks of Ni–Co@Fe–Co PBA are positioned at the same 2θ values as those of Fe–Co PBA. This may be due to the weakening of the diffraction of the Ni–Co PBA core by the Fe–Co PBA shell and the merging induced by their similar lattice constants (Ni–Co PBA: *a* = *b* = *c* = 9.93 Å; Fe–Co PBA: *a* = *b* = *c* = 10.28 Å). Figure 2b displays the Fourier-transform infrared (FT-IR) spectra of NiFe–Co PBA and Ni–Co@Fe–Co PBA. Their main peaks are the same consisting of the O–H stretching mode, H–O–H bending mode, and C≡N stretching mode, which confirm their similar chemical compositions.^19,29,30^ The slight redshift of the C≡N peak in Ni–Co@Fe–Co PBA (2176 cm^–1^) compared with that in NiFe–Co PBA (2190 cm^–1^) indicates the generation of [Co^II^(CN)_6_]^4–^, which weakens the C≡N conjugation and reduces the bond energy.^18^ The small H–O–H peak at 2886 cm^–1^ in Ni–Co@Fe–Co PBA is due to the formation of unstable H–O–H bonds caused by the enhanced water adsorption of the PBA with a core–shell structure.^31^ The STEM–-EDS spectra (Figure 2c) suggest that the N content in Ni–Co@Fe–Co PBA (18.1%) is lower than that in NiFe–Co PBA (24.3%), which is due to the production of {Ni_3_Fe_2_[Co^II^(CN)_6_]}[Co^III^(CN)_6_]_2_ during the encapsulation process. The relative atomic percentages of Ni, Fe, and Co in Ni–Co@Fe–Co PBA and NiFe–Co PBA are basically the same, indicating that they have similar metal element contents (Table S1). The specific surface area and average pore size (Figure 2d,e) of Ni–Co@Fe–Co PBA are 585 m^2^ g^–1^ and 1.95 nm, respectively, which are much larger compared to those of NiFe–Co PBA (220 m^2^ g^–1^, 0.93 nm). XPS was conducted to determine the electronic structure and valence state of the elements in the compounds. The survey scans of the PBA catalysts show that they all contain C, N, O, and Co (Figure S3 and Table S2). Ni–Co@Fe–Co PBA and NiFe–Co have similar atomic percentages of Ni, Fe, and Co, which are basically consistent with the feed ratio. The fitted C 1s edge spectra (Figure 3a) of the PBA-based catalysts reveal two peaks corresponding to C=O and C–C.^32^ The peak ratio of C=O/C–C in trimetallic PBAs is significantly higher than that in bimetallic PBAs, and the distance between the two peaks in Ni–Co@Fe–Co PBA is increased. This may be due to the enhanced water adsorption capabilities caused by the promoted pore structure.^33^ The N 1s edge spectra (Figure 3b) of Ni–Co PBA, Fe–Co PBA, and NiFe–Co PBA are fitted to two peaks assigned to pyrrolic N and pyridinic N, while a new peak corresponding to graphitic N is fitted for Ni–Co@Fe–Co PBA.^34^ The relative total content of pyridinic N and graphitic N significantly increases in trimetallic PBAs (NiFe–Co PBA: 67%, Ni–Co@Fe–Co PBA: 78%) compared to that in bimetallic PBAs (Ni–Co PBA: 54%, Fe–Co PBA: 56%) (Table S3). The pyridinic N and graphitic N are inducive to the adsorption and dissociation of water molecules,^35^ suggesting better water-splitting kinetics for the trimetallic PBAs. Figure 3c shows the two peaks in the Co 2p edge spectra of Ni–Co PBA, Fe–Co PBA, and NiFe–Co PBA are attributed to 2p_1/2_ and 2p_3/2_ spins of Co^3+^, while the additional peak appearing in Ni–Co@Fe–Co PBA is ascribed to the 2p_1/2_ and 2p_3/2_ spins of metallic Co.^36,37^ This may be due to the reduction of [Co^II^(CN)_6_]^4–^ that is induced by the oxidation of excess ferrous ions. The peaks fitted for the Ni 2p edge (Figure 3d) correspond to the 2p_1/2_ and 2p_3/2_ spins of Ni^2+^ and Ni^3+^, and the relative content of Ni^3+^ is the highest in Ni–Co@Fe–Co PBA among the Ni-containing PBAs.^26^Figure 3e displays the Fe 2p edge spectra of the Fe-containing PBAs. It is observed that the relative content of Fe^3+^ in Ni–Co@Fe–Co PBA is significantly increased compared with the other PBAs (Table S3), which is due to the oxidation of ferrous ions during the etching process.^38^ The prominent electron paramagnetic resonance (EPR) signal in Ni–Co@Fe–Co PBA at *g* = 2.005 compared to that of NiFe–Co PBA suggests that [Co^III^(CN)_6_]^3–^ vacancies are generated due to the etching of ferrous ions in the encapsulation process(Figure 3f).^39^Figure 3g and Table S4 display the spider chart showing the structural and compositional parameters for comparison of Ni–Co@Fe–Co PBA and NiFe–Co PBA, which indicates that the rational design has a greater potential to enhance the performance of PBA-based electrocatalysts.

### 2.2 Evaluation of the Electrochemical HER Performance

**Figure 4:**
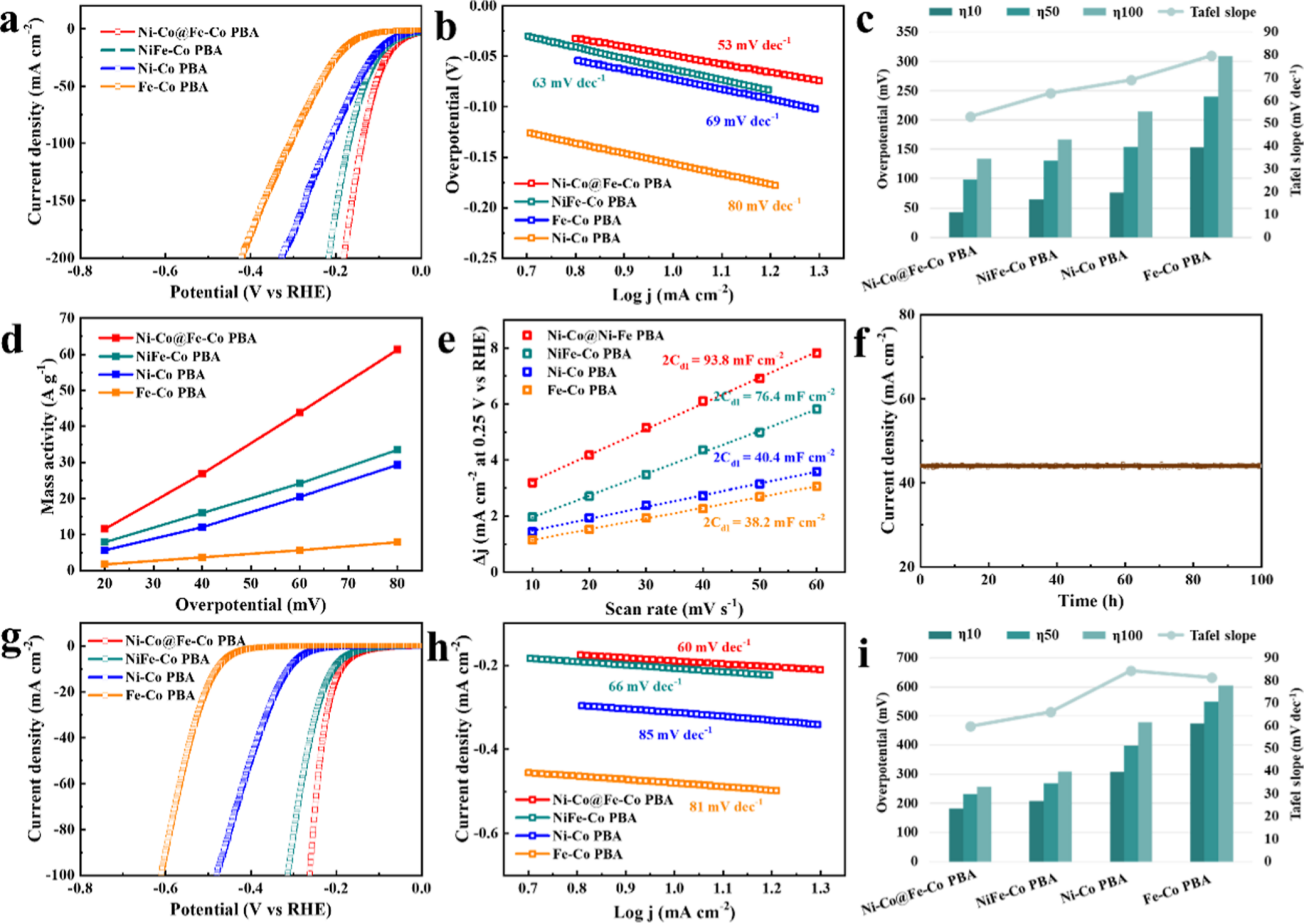
Electrochemical performance for HER. (a) Polarization curves and (b) Tafel plots of Ni–Co@Fe–Co PBA, NiFe–Co PBA, Ni–Co PBA, and Fe–Co PBA in alkaline freshwater (1 M KOH). (c) Tafel slope comparison and the overpotential comparison needed to deliver cathodic current densities of 10, 50, and 100 mA cm^–2^ for Ni–Co@Fe–Co PBA, NiFe–Co PBA, Ni–Co PBA, and Fe–Co PBA in alkaline freshwater. (d) Mass activity of Ni–Co@Fe–Co PBA, NiFe–Co PBA, Ni–Co PBA, and Fe–Co PBA. (e) Capacitive current measured at 0.25 V of Ni–Co@Fe–Co PBA, NiFe–Co PBA, Ni–Co PBA, and Fe–Co PBA as a function of the scan rate. (f) Chronoamperometry measurement of Ni–Co@Fe–Co PBA at an applied potential of −0.1 V vs RHE in alkaline freshwater. (g) Polarization curves and (h) Tafel plots of Ni–Co@Fe–Co PBA, NiFe–Co PBA, Ni–Co PBA, and Fe–Co PBA in alkaline simulated seawater (1 M KOH + 0.5 M NaCl). (i) Tafel slope comparison and the overpotential comparison needed to deliver cathodic current densities of 10, 50, and 100 mA cm^–2^ for Ni–Co@Fe–Co PBA, NiFe–Co PBA, Ni–Co PBA, and Fe–Co PBA in alkaline simulated seawater.

The electrocatalytic performance of the PBA catalysts toward HER was studied by using a standard three-electrode system in 1.0 M KOH. Pt/C was used as the benchmark for reference (Figure S4). As illustrated by the *iR*-compensated linear sweep voltammetry (LSV) curves, the Ni–Co@Fe–Co PBA with a core–shell structure displayed the best activity (Figure 4a,c, Table S5), showing the lowest overpotentials of 43, 99, and 134 mV to reach the current densities of 10, 50, and 100 mA cm^–2^, respectively. The overpotentials of Fe–Co PBA, Ni–Co PBA, and NiFe–Co PBA at the current density of 100 mA cm^–2^ are 309, 215, and 167 mV, respectively. The better HER performance of Ni–Co@Fe–Co PBA is further supported by its smaller Tafel slope of 53 mV dec^–1^ compared with 80 mV dec^–1^ for Fe–Co PBA, 69 mV dec^–1^ for Ni–Co PBA, and 63 mV dec^–1^ for NiFe–Co PBA (Figure 4b,c, Table S5). The obtained Tafel slopes of ∼40 mV dec^–1^ (or slightly higher) suggest that the HER on the PBA surface follows the Volmer–Heyrovsky pathway.^40^ The intrinsic catalytic activity of the PBA samples was evaluated by mass activity and ECSA. The mass activity of Ni–Co@Fe–Co PBA at an overpotential of 60 mV is 43.8 A g^–1^, which is superior to those of Fe–Co PBA (5.7 A g^–1^), Ni–Co PBA (20.4 A g^–1^), and NiFe–Co PBA (24.1 A g^–1^) (Figure 4d). ECSA is proportional to the double-layer capacitance (*C*_dl_), which was calculated from the cyclic voltammetry (CV) curve (Figure S5) and shown in Figure 4e.^4^ The *C*_dl_ of Ni–Co@Fe–Co PBA is 46.9 mF cm^–2^, which is much higher than those of NiFe–Co PBA (38.2 mF cm^–2^), Ni–Co PBA (20.2 mF cm^–2^), and Fe–Co PBA (19.1 mF cm^–2^). Chronoamperometry measurement was used to study the stability of Ni–Co@Fe–Co PBA at an applied potential of −0.1 V versus RHE (Figure 4f) in alkaline freshwater, and there was no obvious degradation after 100 h test. Given the excellent activity and durability in alkaline freshwater (1 M KOH), the HER performance of the PBA catalysts was further tested in alkaline simulated seawater (1 M KOH + 0.5 M NaCl) (Figure 4g–h, Table S6). A similar trend was observed for the PBA samples, and the overpotentials at the current density of 100 mA cm^–2^ and the Tafel slopes for Fe–Co PBA, Ni–Co PBA, and NiFe–Co PBA were 606 mV and 81 mA dec^–1^, 478 mV and 85 mV dec^–1^, and 309 mV and 66 mV dec^–1^, respectively. Ni–Co@Fe–Co PBA showed superior catalytic activity in comparison to the other samples, exhibiting the lowest overpotential (η_10_ = 183 mV, η_50_ = 233 mV, η_100_ = 258 mV) and Tafel slope (60 mV dec^–1^). The Nyquist plots obtained from electrochemical impedance spectroscopy (EIS) of the PBA catalysts are shown in Figure S6, and the charge-transfer resistances (*R*_ct_) of Fe–Co PBA, Ni–Co PBA, NiFe–Co PBA, and Ni–Co@Fe–Co PBA are 16.14, 13.88, 10.69, and 9.48 Ω in 1 M KOH and 32.30, 15.57, 15.03, and 10.08 Ω in 1 M KOH + 0.5 M NaCl, respectively (Tables S7 and S8). This is consistent with the activity trend at −0.050 V versus RHE in alkaline freshwater and −0.200 V versus RHE in alkaline simulated seawater, respectively. The *R*_ct_s of these catalysts in alkaline freshwater are also lower than those in alkaline simulated seawater, indicating their higher efficiencies of electron transfer without the addition of NaCl. The stability of Ni–Co@Fe–Co PBA in alkaline simulated seawater was also tested by chronoamperometry measurement at an applied potential of −0.23 V versus RHE (Figure S7), and the retention rate after 24 h test was 98.4%, which is slightly inferior to the durability in alkaline freshwater. The lower HER activity of the PBA catalysts in alkaline simulated seawater compared to alkaline freshwater is due to Cl^–^-induced chlorine evolution reaction, hypochlorite formation, and chloride corrosion under alkaline conditions.^8,41^

### 2.3 Active Site Identification

**Figure 5:**
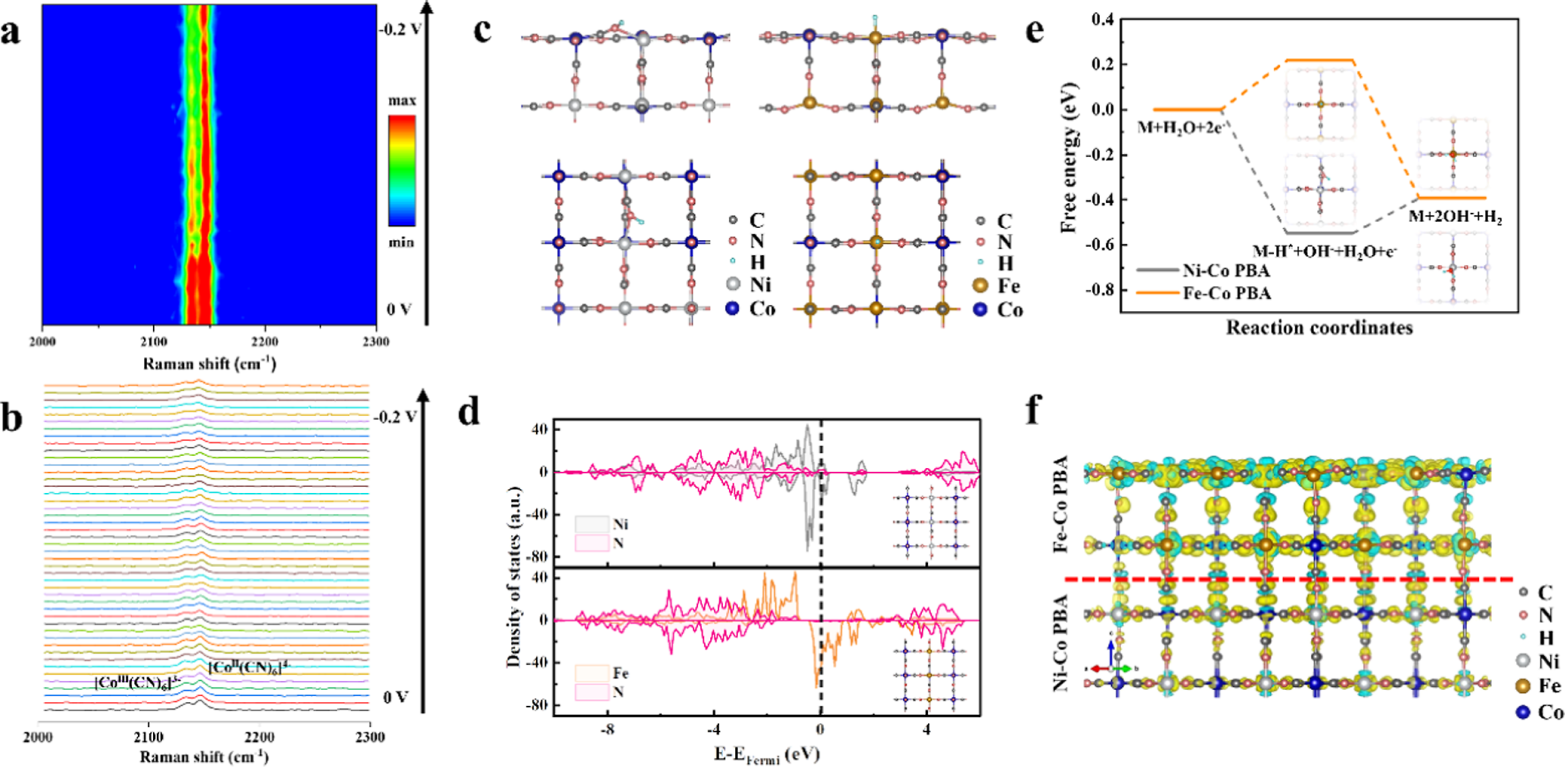
Active site identification. (a) Contour plot and (b) corresponding Operando Raman curves of Ni–Co@Fe–Co PBA obtained from the voltage changing from 0 to −0.2 V (vs RHE) in alkaline freshwater. (c) Optimized adsorption structures of H* on Ni–Co PBA and Fe–Co PBA (top: side view; bottom: top view). (d) DOSs of Ni–Co PBA and Fe–Co PBA (inset: the corresponding optimized structural models, top: Ni–Co PBA, bottom: Fe–Co PBA). (e) Gibbs free energy diagrams of Ni–Co PBA and Fe–Co PBA for HER in alkaline solution. (f) Charge-transfer diagram (yellow and blue isosurfaces indicate the gain and loss of electrons) of Ni–Co@Fe–Co PBA at the junction of NiCo-PBA and Fe–Co PBA.

Postmortem characterizations were conducted to shed light on the catalytically active sites for high HER activity of the Ni–Co@Fe–Co PBA catalyst. The microstructure change was revealed by the TEM images (Figure S8). There is no obvious fragmentation and collapse observed after a long-term durability test, indicating strong structural stability. XRD was performed after the test in alkaline simulated seawater (1 M KOH + 0.5 M NaCl) to study the change of crystal structure, and it is found that the peak position did not shift in the XRD pattern of Ni–Co@Fe–Co PBA after HER, while a broad peak appeared around 20°, indicating that amorphization occurred during the HER process (Figure S9). The evolution of chemical composition and valence state was detected by operando Raman spectroscopy (alkaline freshwater) and XPS (alkaline simulated seawater). The two peaks at 2134 and 2145 cm^–1^ in the Raman spectra are attributed to the stretching vibrations of C≡N and denoted as peak A and peak B, respectively (Figure 5a,b), which are sensitive to the oxidation states of coordinated cobalt ions (Co^III^ and Co^II^, respectively).^4,41,42^ It is observed that the overall intensity of the peak decreases slightly with the applied potential, indicating some loss of catalyst. The intensity of peak A significantly decreases compared to that of peak B, suggesting that Co^3+^ is reduced. The XPS survey scan and high-resolution spectra of Ni–Co@Fe–Co PBA after the long-term stability test in alkaline simulated seawater (1 M KOH + 0.5 M NaCl) are shown in Figure S10. The peak positions and relative contents of chemical bonds and valence of deconvoluted C 1s, N 1s, Co 2p, Ni 2p, and Fe 2p edges for post-HER Ni–Co@Fe–Co PBA are listed in Table S9. The relative peak intensities of metal elements (Co, Ni, and Fe) in Ni–Co@Fe–Co PBA after HER are lower than those in the fresh Ni–Co@Fe–Co PBA (Figure S10a), which may be due to hypochlorite formation and chloride corrosion. The high-resolution C 1s edge spectrum of Ni–Co@Fe–Co PBA after HER indicates the formation of C–O–C bonds due to the adsorbed water on the surface (Figure S10b). The same three peaks were fitted in the N 1s edge spectrum, while the content of graphite nitrogen is reduced (Figure S10c) compared to that of the fresh Ni–Co@Fe–Co PBA. From the Co 2p edge spectrum, the content of metallic Co increases, revealing that Co^3+^ is reduced during the HER process (Figure S10d), which is consistent with the operando Raman spectroscopy results. Similar results were also observed from the Ni 2p and Fe 2p edge spectra of Ni–Co@Fe–Co PBA after HER (Figure S10e,f). The contents of Ni^2+^ and Fe^2+^ increase, while those of Ni^3+^ and Fe^3+^ decrease, demonstrating that Ni and Fe elements were reduced under the reduction potential. The disappearance of the satellite peaks in the Ni 2p edge spectrum suggests that the remaining Ni after HER mainly exists in the far-surface area.

Theoretical simulations were performed to understand the HER activities of the PBA-based catalysts. The optimized adsorption structures of H* on Ni–Co PBA and Fe–Co PBA are shown in Figure 5c. It is observed that the H* intermediates have different preferred adsorption sites in the PBA samples, forming H*–N bonds in Ni–Co PBA and H*–Fe bonds in Fe–Co PBA, respectively. This can be explained by the density of state (DOS) in Figure 5d. Compared with the Fe 3d band, the Ni 3d band has more overlap with the N 2p band, leading to better covalency of the Ni–N bond, which enables the H* intermediate preferentially binding to the adjacent N. Figure 5e shows the free-energy diagrams of Ni–Co PBA and Fe–Co PBA for HER in alkaline solution. The free-energy differences for the Volmer step of Ni–Co PBA and Fe–Co PBA are −0.548 and 0.218 eV, respectively, and the free-energy differences for the Heyrovsky step of Ni–Co PBA and Fe–Co PBA are 0.155 and −0.611 eV, respectively. Hence, the energy barriers for the Volmer–Heyrovsky pathway on Ni–Co PBA and Fe–Co PBA in the HER process are 0.155 and 0.218 eV, respectively. The lower energy barrier of Ni–Co PBA suggests a better HER activity than Fe–Co PBA, which is consistent with the experimental results. Furthermore, the negative free-energy difference of the Volmer step of Ni–Co PBA implies that the H*–N adsorption sites are more favorable for HER activity than the H*–Fe sites in Fe–Co PBA. The comparison of the total DOS of NiFe–Co PBA and Ni–Co PBA (Figure S11) shows that when Fe species are introduced into the structure, the intensity of states near the Fermi level is higher, thereby increasing the electron mobility. The modeled structures of Ni–Co@Fe–Co PBA and NiFe–Co PBA are shown in Figures S12 and S13, respectively. Ni–Co@Fe–Co PBA and NiFe–Co PBA possess the same adsorption sites as Ni–Co PBA due to similar band structures. According to the results of Bader charge analysis (Figure 5f), the partial charges of N atoms on the Ni–Co side of Ni–Co@Fe–Co PBA, NiFe–Co PBA, and Ni–Co PBA are −1.22, −1.24, and −1.16 e, respectively. The large number of electrons on N atoms on the Ni–Co side lowers the energy barrier in Ni–Co@Fe–Co PBA,^43^ leading to the best HER activity among the materials. The charge-density analysis (Figure 5f) at the Ni–Co@Fe–Co PBA interface implies that there is an electron transfer from the Fe–Co PBA side to the Ni–Co PBA side. This makes the neighboring N of Ni at the interface more negatively charged than N inside the Ni–Co PBA side, which generates more H* intermediates on the interface, thereby enhancing the HER activity.^44^

### 2.4 Electrochemical Evaluation on Seawater Splitting

**Figure 6:**
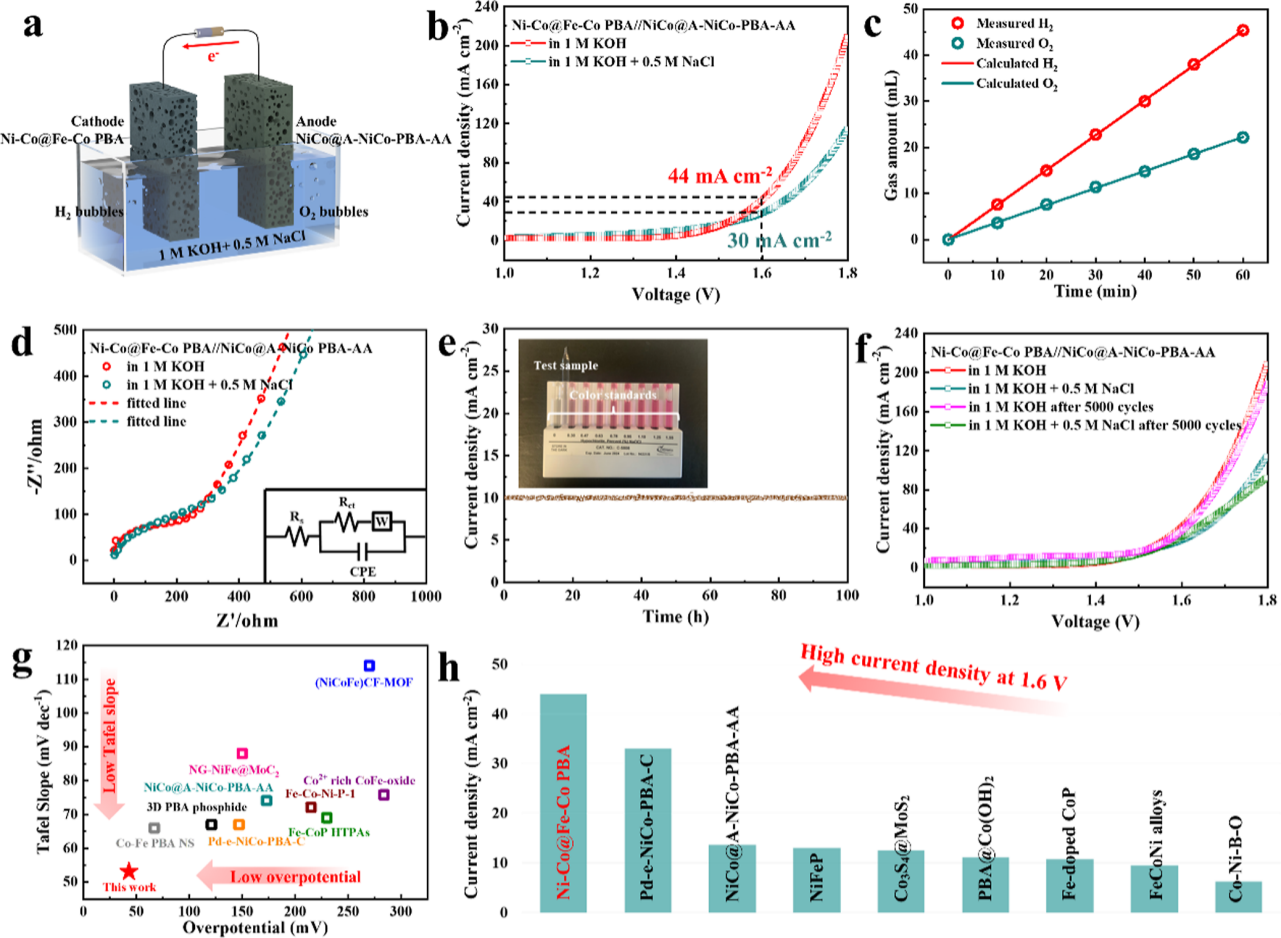
Seawater-splitting performance. (a) Schematic illustration of the water-splitting electrolyzer. (b) Overall water-splitting performance of the Ni–Co@Fe–Co PBA//NiCo@A-NiCo-PBA-AA electrode couple in alkaline freshwater and alkaline simulated seawater. (c) Detected and calculated gaseous product (H_2_ and O_2_) amounts by the electrolyzer at a fixed current density of 50 mA cm^–2^. (d) Nyquist plots of Ni–Co@Fe–Co PBA//NiCo@A-NiCo-PBA-AA (inset: equivalent electric circuit). (e) Chronoamperometry stability test for the Ni–Co@Fe–Co PBA//NiCo@A-NiCo-PBA-AA electrode couple at a voltage of 1.47 V. (f) Overall water-splitting performance of the Ni–Co@Fe–Co PBA//NiCo@A-NiCo-PBA-AA electrode couple before and after 5000 cycles. Comparison of (g) HER and (h) water-splitting performance with some recently reported PBA-derived catalysts in the alkaline electrolyte.

To utilize the excellent hydrogen evolution performance of the material, a water-splitting electrolyzer was set up for overall alkaline freshwater (1 M KOH) and simulated alkaline seawater (1 M KOH + 0.5 M NaCl) splitting, as illustrated in Figure 6a. The Ni–Co@Fe–Co PBA electrode (cathode) was coupled with our previously reported OER catalyst of NiCo@A-NiCo-PBA-AA (a crystalline Ni–Co PBA with an amorphous Ni–Co PBA shell after activation, anode),^19^ and nickel foams were used as the substrates (Figure S14). The electrolyzer provided high current densities of 44 and 30 mA cm^–2^ at a cell voltage of 1.6 V in alkaline freshwater and simulated seawater splitting, respectively, suggesting good activity (Figure 6b). The generated O_2_ and H_2_ gases were collected to calculate the Faradaic efficiency (Figure 6c). At steady state, the H_2_/O_2_ evolution rate detected had a molar ratio approaching 2/1, and the measured gas amounts were in good agreement with the theoretical values, demonstrating a nearly 100% Faradaic efficiency of the Ni–Co@Fe–Co PBA//NiCo@A-NiCo-PBA-AA electrolyzer. EIS was performed to measure the interfacial resistance of the corresponding process in both alkaline freshwater and simulated alkaline seawater (Figure 6d), and the inset of Figure 6d shows the equivalent electric circuit. It is found that the Ni–Co@Fe–Co PBA//NiCo@A-NiCo-PBA-AA electrode pair exhibits a lower charge-transfer resistance (*R*_ct_) in freshwater (12.31 Ω) compared with that in simulated seawater (15.72 Ω), revealing the better charge-transfer kinetics. The comparable series resistances (*R*_s_) in freshwater and simulated seawater may be because the ionic concentration in both solutions is above 1 M, and the *R*_s_ is relatively stable with the increase of ion concentrations in this range.^45^ The stability of the Ni–Co@Fe–Co PBA//NiCo@A-NiCo-PBA-AA electrode couple in alkaline simulated seawater was tested by chronoamperometry measurement (Figure 6e). No obvious attenuation was observed after 100 h test. The *N*,*N*-diethyl-*p*-phenylenediamine (DPD) method was conducted to examine the possible formation of Cl-oxidation products (ClO^–^) in the measurement (Figure 6e, inset).^46^ DPD reacts with hypochlorite ions and/or hypochlorous acid to yield a pink color, correlating quantitatively with the concentration of these species in solution. The result showed that no pink color was produced, indicating that no hypochlorite ion or hypochlorous acid was formed in the reaction. The water-splitting performance of the Ni–Co@Fe–Co PBA//Ni–Co@Fe–Co PBA electrode couple was also tested for comparison, and it was significantly inferior to that of the Ni–Co@Fe–Co PBA//NiCo@NiCo-PBA-AA electrode couple (Figure S15).

The CV measurements were conducted in 1 M KOH at room temperature from 1 to 1.8 V with a scan rate of 50 mV s^–1^ to further validate the stability of the catalyst. The LSV comparison (Figure 6f) of water splitting before and after 5000 cycles of CV shows that the Ni–Co@Fe–Co PBA//NiCo@A-NiCo-PBA-AA electrode pair exhibited only slight decay, which is consistent with the results of the chronoamperometry test, demonstrating its robust durability. The remarkable HER activity of Ni–Co@Fe–Co PBA and the water-splitting activity of the Ni–Co@Fe–Co PBA//NiCo@A-NiCo-PBA-AA electrode pair are superior to most of the PBA-derived HER electrocatalysts and water-splitting electrode couples previously reported in alkaline freshwater (Figure 6g,h and Tables S10 and S11) and are comparable to the state-of-the-art non-noble metal-based HER electrocatalysts and water-splitting electrode couples previously reported in alkaline seawater (Tables S12 and S13).

## 3 Conclusions

In summary, a trimetallic core–shell PBA nanobox HER electrocatalyst was prepared by an iterative strategy involving coprecipitation and chemical etching processes. Owing to the porous core–shell structure and trimetallic composition, the optimized Ni–Co@Fe–Co PBA delivers superior HER performance, with overpotentials of 43 and 183 mV at 10 mA cm^–2^ and Tafel slopes of 53 and 60 mV dec^–1^ in alkaline freshwater and simulated seawater, respectively. Operando Raman spectroscopy reveals the evolution from Co^3+^ to Co^2+^ which contributes to the active sites. DFT calculations indicate that the formation of H*–N adsorption sites and the introduction of Fe species reduce the energy barrier of HER, and electrons are transferred from the Fe–Co PBA side to the Ni–Co PBA side at the Ni–Co@Fe–Co PBA interface, resulting in the generation of H* intermediates, which greatly enhance its HER activity. Furthermore, the fabricated Ni–Co@Fe–Co PBA//NiCo@A-NiCo-PBA-AA electrolyzer delivers high current densities of 44 and 30 mA cm^–2^ to reach 1.6 V in alkaline freshwater and simulated seawater, respectively, exhibiting no obvious attenuation over a 100 h test. This work may inspire the rational design and performance regulation of PBA-based electrocatalysts and extend their applications to other energy-related fields.

## Supporting Information Available

Materials and synthesis methods; characterization methods; electrochemical measurements; DFT calculations; additional structural and chemical composition characterizations (SEM, TEM, XPS, and XRD); and additional electrochemical performance (PDF)
