# Supplementary material for: Heterostructured Core–Shell Ni–Co@Fe–Co Nanoboxes of Prussian Blue Analogues for Efficient Electrocatalytic Hydrogen Evolution from Alkaline Seawater
Source: ACS Catal. 2023 Jan 9;13(2):1349–58. doi: 10.1021/acscatal.2c05433 (PMC9872088; doi:10.1021/acscatal.2c05433)
Supplement: Supplementary file 1 — cs2c05433_si_001.pdf [file cs2c05433_si_001.pdf]

Supporting Information

Heterostructured Core-Shell Ni-Co@Fe-Co Nanoboxes of Prussian Blue Analogues for Efficient Electrocatalytic Hydrogen Evolution from Alkaline Seawater

Hao Zhang[1], Jiefeng Diao[2], Mengzheng Ouyang[3], Hossein Yadegari[1], Mingxuan Mao[4], Mengnan Wang[1], Graeme Henkelman[2], Fang Xie[1], D. Jason Riley[1,*]

[1]Department of Materials and London Center for Nanotechnology, Imperial College London, London, SW7 2AZ, UK. E-mail: jason.riley@imperial.ac.uk

[2]Department of Chemistry and the Oden Institute for Computational Engineering and Sciences, The University of Texas at Austin, Austin, TX, 78712 USA.

[3]Department of Earth Science and Engineering, Imperial College London, London, SW7 2AZ UK.

[4]Department of Electrical and Electronic Engineering, Imperial College London, London, SW7 2AZ UK.

Experimental section

Materials and methods

Materials: The following reagents were obtained and used as received without further processing: nickel(II) sulfate hexahydrate (≥ 98.5%), iron(II) sulfate heptahydrate (> 98.5%), sodium citrate dihydrate (≥ 99.0%) and potassium hexacyanocobaltate(III) (> 99.0 %) were bought from Sigma-Aldrich. Reverse Osmosis water (> 18.2 MOhms cm^{-1}) was utilized in all experimental procedures.

Preparation of Ni-Co PBA: Solution A was prepared by dissolving 0.06 mmol nickel sulfate hexahydrate and 0.09 mmol sodium citrate dihydrate in 5 mL deionized water. Solution B was prepared by dissolving 0.04 mmol of potassium hexacyanocobaltate(III) in 5 mL of deionized water. Then, solution B was added to solution A dropwise under magnetic stirring for 10 min, and the resultant solution aged for 24 h at room temperature. The powdery product was collected by centrifugation, washed several times with deionized water, and vacuum dried at 60 °C overnight.

Preparation of Fe-Co PBA: The same procedures as for the preparation of Ni-Co PBA were followed, except that 0.06 mmol nickel sulfate hexahydrate was replaced with 0.06 mmol iron(II) sulfate heptahydrate.

Preparation of NiFe-Co PBA: The same procedures as the preparation of Ni-Co PBA were followed, except that 0.06 mmol nickel sulfate hexahydrate was replaced with a solution containing 0.03 mmol nickel sulfate hexahydrate and 0.03 mmol iron(II) sulfate heptahydrate.

Preparation of Ni-Co@Fe-Co PBA: Solution A was prepared by adding 10 mg Ni-Co PBA and dissolving 0.06 mmol iron(II) sulfate heptahydrate and 0.09 mmol sodium citrate dihydrate in 5 mL deionized water. Solution B was prepared by dissolving 0.04 mmol of potassium hexacyanocobaltate(III) in 5 mL of deionized water. Then, solution B was added to solution A dropwise under magnetic stirring for 10 min, and the resultant solution aged for 24 h at room temperature. The powdery product was collected by centrifugation, washed several times with deionized water, and vacuum dried at 60 °C overnight.

Preparation of NiCo@A-NiCo-PBA-AA (comparison): NiCo@A-NiCo-PBA was activated to obtain NiCo@A-NiCo-PBA-AA. The preparation of NiCo@A-NiCo-PBA refers to the method previously reported by our group.[1] The activation of NiCo@A-NiCo-PBA was performed at an applied potential of 1.55 V (vs. RHE) for 10 min. A NiCo@A-NiCo-PBA-coated FTO glass served

as the substrate for the working electrode. A Pt mesh and saturated Ag/AgCl/Cl^- were employed as the counter electrode and reference electrode, respectively. An aqueous solution of 1 M NaOH was the electrolyte. After the activation, the working electrode was taken out from the electrolyte and dried at room temperature. NiCo@A-NiCo-PBA-AA was scratched off the FTO substrate as the anode material for water splitting.[1]

Characterizations

Powder X-ray diffraction (XRD) patterns were collected on a Bruker D2 ADVANCE diffractometer with Cu K_α radiation (λ=1.5418Å). The structure and morphology of the samples were characterized by field-emission scanning electron microscopy (FESEM, Zeiss LEO 1525) and transmission electron microscopy (TEM, JEOL-2100Plus, JEOL-2100F). Energy-dispersive X-ray spectroscopy (EDS) attached to the TEM was used to analyze the composition of the nanoscale samples. Fourier-transform infrared (FT-IR) spectra were collected on a Thermo Scientific Nicolet iS50 FT-IR spectrometer fitted with a diamond ATR module, 4000-400 cm^{-1}, 64 scans, and 0.5 cm^{-1} resolution. The pore structures of samples were characterized using the N_2 adsorption/desorption isotherm tested on the Micromeritics 3 Flex Physisorption at 77 K. The specific surface area was determined by the multi-point Brunauer-Emmett-Teller (BET) method and the pore-size distribution was calculated based on the Barrett-Joyner-Halenda (BJH) method. X-ray photoelectron spectroscopy (XPS) analysis was conducted on a PHI-5000 VersaProbe X-ray photoelectron spectrometer using an Al K_α X-ray source. The powder samples were stuck onto the specific sample holders using conductive double-sided carbon tapes for the test. Continuous wave electron paramagnetic resonance (EPR) spectra were recorded at X-band (*ca.* 9 GHz) on a Bruker EMX Micro spectrometer equipped with a Bruker ER4112SHQ resonator at room temperature. Samples were placed in quartz EPR tubes (4 mm OD, 3 mm ID) in identical quantities, placed at the same optimal position in the cavity and measured under non-saturating conditions. G values were obtained by comparison with a Bruker Strong Pitch standard (g = 2.0028). *Operando* Raman spectra were obtained with an inVia Renishaw confocal Raman microscope operated with an incident laser beam at 532 nm focused through a 50x objective (Leica). The laser intensity was set to < 1 mW and Raman spectra were collected in static mode, with an exposure time of a few seconds every 5 minutes to minimize sample heating. To monitor the evolution of catalyst samples during HER

process in alkaline freshwater, each Raman spectrum was collected after a constant potential was applied to the catalyst electrode for 5 min. Each Raman spectrum was obtained using an integration time of a few seconds, accumulating 5 times. The laser shutter remained closed between spectrum collections.

Electrochemical measurements

The electrochemical tests of the materials were performed using a Metrohm Autolab electrochemical workstation PGStat-12 (Utrecht, the Netherlands) connected to a three-electrode cell. A glassy carbon electrode (GCE) of 3 mm diameter served as the substrate for the working electrode, and a graphite rod and a $Hg/HgO/OH^-$ electrode were employed as the counter electrode and reference electrode, respectively. 5 mg of the as-prepared PBA catalyst was dispersed in 4.5 mL of a water/isopropanol solution (1:3) containing 500 μL Nafion (5%). The resulting solution was sonicated for 0.5-1 h. When the solution was well dispersed, 4 μL of the above solution was dropped onto the clean GCE for electrochemical studies.

Ar-saturated aqueous solutions of alkaline freshwater (1 M KOH) and simulated alkaline seawater (1 M KOH + 0.5 M NaCl) were the electrolytes for HER experiments. Cyclic voltammetry (CV) curves were recorded at a sweep rate of 100 mV s^{-1} for multiple cycles. Linear sweep voltammetry (LSV) was carried out at a scan rate of 5 mV s^{-1} for polarization curves. LSV was performed several times until the signals were stabilized. Nyquist plots on Ni-Co PBA, Fe-Co PBA, NiFe-Co PBA and Ni-Co@Fe-Co PBA were recorded at -0.050 V vs. RHE in 1 M KOH and at -0.200 V vs. RHE in 1 M KOH + 0.5 M NaCl by applying an AC voltage of 5 mV amplitude in a frequency range from 10 kHz to 0.01 Hz. CV curves with different scan rates (10-60 mV s^{-1}) were measured over a potential range in which redox processes were absent to calculate the electrochemical double-layer capacitance: $C_{dl} = (j_a - j_c)/(2v) = (j_a + |j_c|)/(2v) = \Delta j/(2v)$, where C_{dl} is the double-layer capacitance (F cm^{-2}) of the electroactive materials, j_a and j_c is the anodic and cathodic current density (mA cm^{-2}), respectively, recorded at the middle of the selected potential range, and v is the scan rate (mV s^{-1}). All results reported in this work were converted to the RHE scale according to the Nernst equation,

$$E_{RHE} = E_{Hg/HgO/OH^-} + 0.059 \times \mathrm{pH} + E^0_{Hg/HgO/OH^-}$$

where E_{RHE} is the measured potential referred to RHE, $E_{Hg/HgO/OH^-}$ is the working potential, and

$E^0_{Hg/HgO/OH^-}$ equals to 0.098 V at 25 °C.

The HER activity was obtained after the *i*R-correction to the LSV. Typically, the *i*R-correction is according to the following equation,

$$E = E_{RHE} - 90\% iR$$

where E is the potential after *i*R-correction, E_{RHE} is the measured potential referred to RHE, i represents the measured current, and R is the uncompensated resistance which could be determined by electrochemical impedance spectroscopy (EIS). The uncompensated resistance is found as the real impedance where the imaginary part of the impedance is zero in a Nyquist plot.

A water-splitting device with a two-electrode configuration was assembled. The cathode and the anode electrodes were made by depositing Ni-Co@Fe-Co PBA and NiCo@A-NiCo-PBA-AA onto Ni foam (2×1 cm^2) and then drying in air. To obtain a total catalyst loading of approximately 1 mg cm^{-2}, the deposition process was repeated several times. Then, the Ni foams loaded with catalysts were used as both cathode and anode for water electrolysis.

Computational details

Density functional theory (DFT) calculation was performed using the generalized gradient approximation (GGA) Perdew-Burke-Ernzerhof (PBE) functional, and the projected augmented plane-wave method implemented in the Vienna *ab initio* simulation program (VASP) software code. The Ni-Co PBA and Fe-Co PBA structures were modeled based on the standard unit cell structures of $Ni_3[Co(CN)_6]_2$ (JCPDS card No. 22-1184) and $Fe_3[Co(CN)_6]_2$ (JCPDS card No. 46-0907), respectively. The NiFe-Co PBA structure was modeled based on the Ni-Co PBA structure in which half of the Ni atoms were substituted by Fe atoms, and Ni atoms and Fe atoms were evenly distributed in the system. The Ni-Co@Fe-Co PBA structure was only modeled as the interfacial region, half of which used the Ni-Co PBA model and the other half used the Fe-Co PBA model.

According to the Volmer-Heyrovsky mechanism for HER in alkaline solution, the reaction happening on the PBA catalysts can be written as:

$$PBA + 2H_2O + 2e^- \rightarrow PBA\text{-}H^* + OH^- + H_2O + e^- \rightarrow PBA + 2OH^- + H_2$$

The initial system consists of a PBA molecule, two free H_2O molecules and two extra electrons. The intermediate system consists of a PBA molecule bound to H^*, a free OH^-, a free H_2O molecule and an extra electron. The final system consists of a PBA molecule, two free OH^- and a H_2 molecule.

The Volmer step barrier is the energy difference between the initial system and the intermediate system, and the Heyrovsky step barrier is the energy difference between the intermediate system and the final system.

The Gibbs free energy of the adsorbed intermediate can be calculated as:

$$\Delta G = E_{ads} + E_{PBA} + E_{adsorbate} - T\Delta S$$

where E_{ads} is the adsorption energy of intermediate, E_{PBA} is the energy of different PBA structures, $E_{adsorbate}$ is energy of adsorbate in gas phase, T is the temperature (300 K), ΔS is the entropy various between the adsorption and gas phase. In DFT calculation, the plane wave basis set cutoffs of the wavefunctions were set at 400 eV and zero damping DFT-D3 method was used to investigate weak intermolecular interactions. All the energy data collected were after full relaxation of the system until it had forces on each atom less that 0.01 eV/Å and with an electron step convergence criterion of 10-6 eV. Structures and charge transfer were draw with VESTA and Bader charge was calculated with Bader charge analysis program.

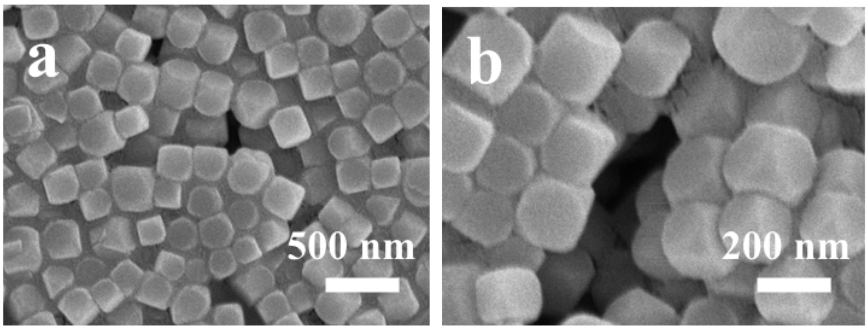

Figure S1. FESEM images of Ni-Co PBA.

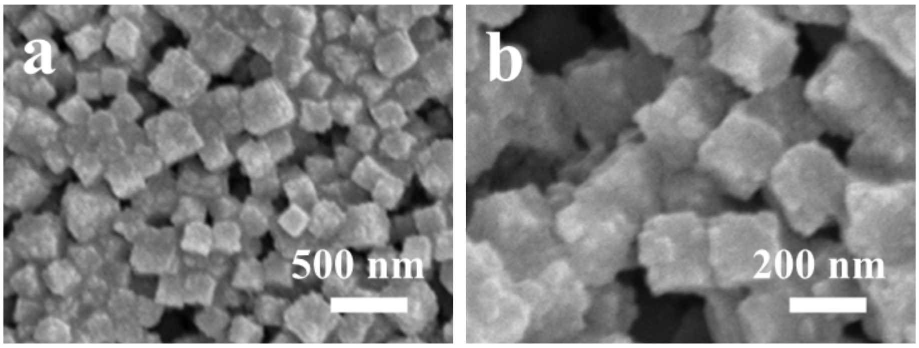

Figure S2. FESEM images of Fe-Co PBA.

Table S1. The atomic relative contents of Ni, Co and Fe in Ni-Co@Fe-Co PBA and NiFe-Co PBA based on their EDS spectra.

	**Ni (%)**	**Fe (%)**	**Co (%)**
**Ni-Co@Fe-Co PBA**	**36.4**	**30.2**	**33.4**
**NiFe-Co PBA**	**36.8**	**28.7**	**34.5**

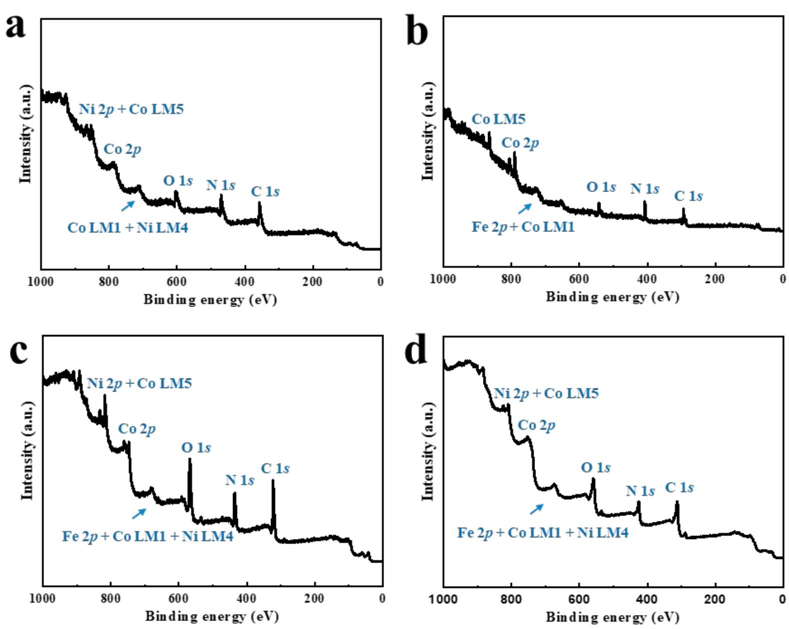

Figure S3. XPS survey scans of (a) Ni-Co PBA, (b) Fe-Co PBA, (c) NiFe-Co PBA and (d) Ni-Co@Fe-Co PBA.

Table S2. Atomic percentages of the surface compositions for Ni-Co PBA, Fe-Co PBA, NiFe-Co PBA and Ni-Co@Fe-Co PBA based on their XPS survey scans.

	C (%)	N (%)	O (%)	Co (%)	Ni (%)	Fe (%)
Ni-Co PBA	28.2	27.7	23.1	8.9	12.0	0.1
Fe-Co PBA	27.8	29.9	23.4	8.1	0	10.8
NiFe-Co PBA	29.3	24.1	27.1	7.9	6.4	5.2
Ni-Co@Fe-Co PBA	29.1	23.8	27.7	7.7	6.1	5.6

Table S3. The peak positions and relative contents of chemical bonds and valence of deconvoluted C 1*s*, N 1*s*, Co 2*p*, Ni 2*p* and Fe 2*p* edges for Ni-Co PBA, Fe-Co PBA, NiFe-Co PBA and Ni-Co@Fe-Co PBA.

Catalysts		Ni-Co PBA		Fe-Co PBA		NiFe-Co PBA		Ni-Co@Fe-Co PBA	
		B.E. (eV)	Area (%)	B.E. (eV)	Area (%)	B.E. (eV)	Area (%)	B.E. (eV)	Area (%)
C 1*s*	C=O	289.1	24	289.2	25	289.1	32	289.4	63
	C-C	285.1	76	285.2	75	285.2	68	284.8	37
N 1*s*	Pyrrolic N	398.7	46	398.7	44	398.9	33	398.9	22
	Pyridinic N	398.3	54	398.4	56	398.2	67	398.2	62
	Graphite N	/	/	/	/	/	/	396.5	16
Co $2p_{1/2}$	Co^{3+}	797.6	38	797.6	42	797.9	43	797.9	20
	Co^0	/	/	/	/	/	/	796.2	21
Co $2p_{3/2}$	Co^{3+}	782.6	62	782.6	58	782.9	57	782.9	35
	Co^0		/	/	/	/	/	781.2	24
Ni $2p_{1/2}$	Ni^{3+}	881.3	13	/	/	881.3	19	881.5	22
	Ni^{2+}	879.4	22	/	/	879.5	18	879.3	18
Ni $2p_{3/2}$	Ni^{3+}	863.4	27	/	/	863.4	30	863.4	32
	Ni^{2+}	861.9	48	/	/	861.9	33	861.9	28
Fe $2p_{1/2}$	Fe^{3+}	/	/	727.0	15	727.4	16	728.2	21
	Fe^{2+}	/	/	725.2	27	725.2	26	725.4	24
Fe $2p_{3/2}$	Fe^{3+}	/	/	714.9	25	714.9	25	716.2	25
	Fe^{2+}	/	/	712.1	33	712.1	33	712.4	30

Table S4. The structural and compositional parameters of Ni-Co@Fe-Co PBA and NiFe-Co PBA.

	$d_{(200)}$ (nm)	$FWHM_{(200)}$ (°)	S_{BET} (m^2 g^{-1})	D_P (nm)	W_{Fe} (%)	1-$W_{Pyrrolic\ N}$ (%)
Ni-Co@Fe-Co PBA	5.12	0.57	585	1.95	13.7	78
NiFe-Co PBA	5.01	0.42	220	0.93	12.1	67

$\mathbf{d_{(200)}}$: calculated average interlayer spacing of (200) planes from XRD patterns.

$\mathbf{FWHM_{(200)}}$: fitted full width at half maximum of (200) peaks from XRD patterns.

$\mathbf{S_{BET}}$: specific surface areas calculated using the multipoint Brunauer-Emmett-Teller (BET) method.

$\mathbf{D_p}$: average pore diameter from N_2 adsorption and desorption isotherms based on the (non-local density functional theory) method.

$\mathbf{W_{Pyrrolic\ N}}$: The percentages of Pyrrolic N obtained by XPS analysis.

Note: The disorder degree was determined by the full width at half maximum (FWHM) of the strongest diffraction peak in the XRD pattern and the interlayer spacing of the corresponding crystal plane. The strongest peak corresponds to the (200) plane, and the FWHM of the (200) plane in Ni-Co@Fe-Co PBA and NiFe-Co PBA were 0.57° and 0.42°, respectively, and their ratio was 1:0.737. The increase in interlayer spacing of the (002) plane in Ni-Co@Fe-Co PBA compared to that in NiFe-Co PBA also indicated an increase in disorder degree.

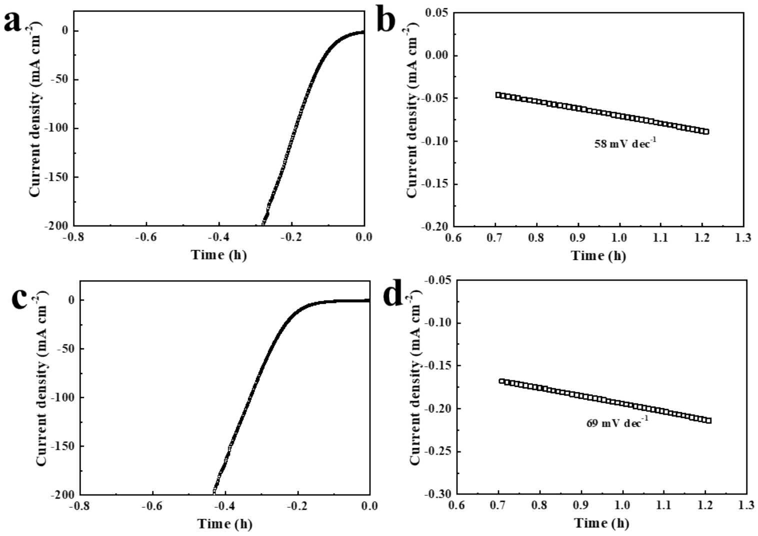

Figure S4. (a) HER polarization curve and (b) HER Tafel plot of Pt/C in alkaline freshwater (1 M KOH). (c) HER polarization curve and (d) HER Tafel plot of Pt/C in alkaline simulated seawater (1 M KOH + 0.5 M NaCl).

The η_{10} overpotentials and Tafel slopes of Pt/C in alkaline freshwater and simulated seawater were 68 mV and 58 mV dec^{-1}, and 194 mV and 69 mV dec^{-1}, respectively. This showed that the activity of Pt/C was lower than that of Ni-Co@Fe-Co PBA, and was comparable to that of NiFe-Co PBA in the two alkaline electrolytes.

Table S5. The Tafel slope and the overpotential comparisons of Ni-Co@Fe-Co PBA, NiFe-Co PBA, Ni-Co PBA and Fe-Co PBA for electrochemical HER in alkaline freshwater (1 M KOH).

	η_{10} HER (mV)	η_{50} HER (mV)	η_{100} HER (mV)	Tafel Slope (mV dec^{-1})
Ni-Co@Fe-Co PBA	43	99	134	53
NiFe-Co PBA	64	131	167	63
Ni-Co PBA	76	154	215	69
Fe-Co PBA	153	240	309	80

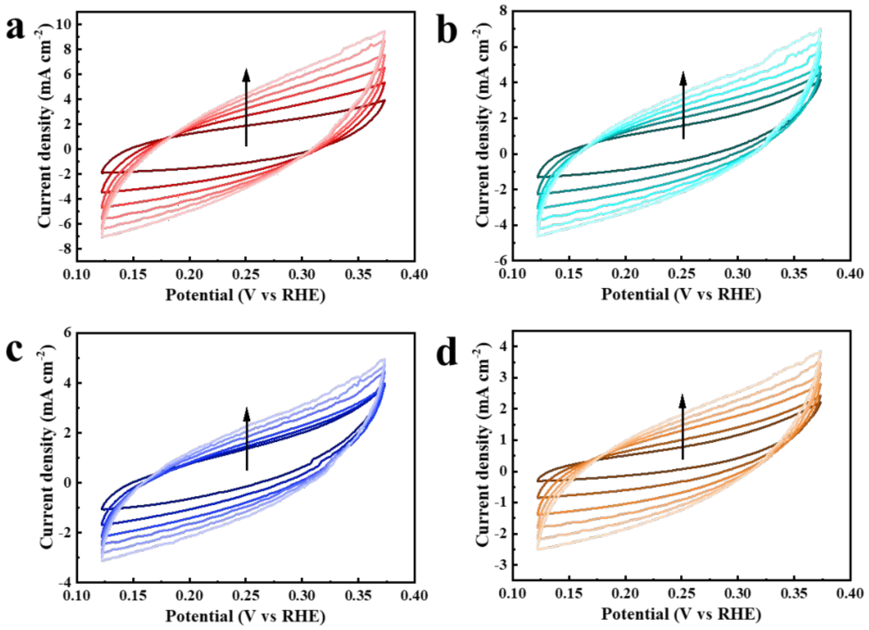

Figure S5. CV curves of (a) Ni-Co@Fe-Co PBA, (b) NiFe-Co PBA, (c) Ni-Co PBA and (d) Fe-Co PBA at different scan rates (10-60 mV s^{-1}) in alkaline freshwater (1 M KOH).

Table S6. The Tafel slope and the overpotential comparisons of Ni-Co@Fe-Co PBA, NiFe-Co PBA, Ni-Co PBA and Fe-Co PBA for electrochemical HER in alkaline simulated seawater (1 M KOH + 0.5 M NaCl).

	η_{10} HER (mV)	η_{50} HER (mV)	η_{100} HER (mV)	Tafel Slope (mV dec^{-1})
Ni-Co@Fe-Co PBA	183	233	258	60
NiFe-Co PBA	208	269	309	66
Ni-Co PBA	307	397	478	85
Fe-Co PBA	474	550	606	81

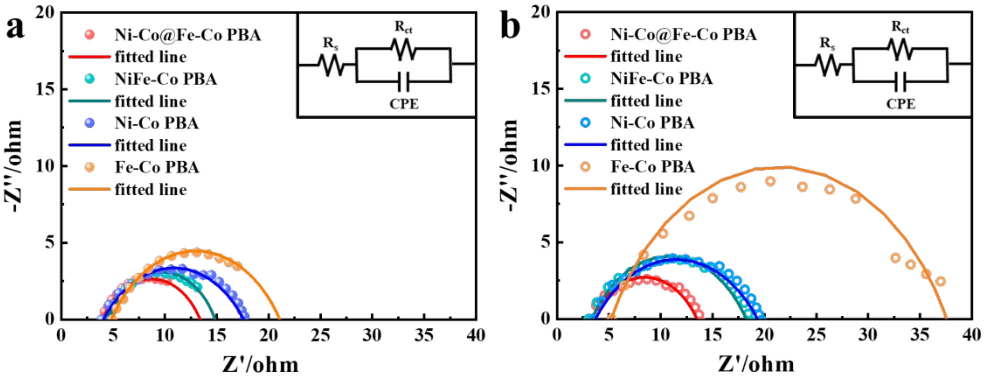

Figure S6. Nyquist plots of Fe-Co PBA, Ni-Co PBA, NiFe-Co PBA and Ni-Co@Fe-Co PBA in a three-electrode configuration recorded (a) at -0.050 V vs. RHE in alkaline freshwater (1 M KOH) and (b) at -0.200 V vs. RHE in alkaline simulated seawater (1 M KOH + 0.5 M NaCl) (inset: equivalent electric circuits).

Table S7. The value and error of each component in the equivalent circuit for Fe-Co PBA, Ni-Co PBA, NiFe-Co PBA and Ni-Co@Fe-Co PBA in a three-electrode configuration in alkaline freshwater (1 M KOH).

	R_s (Ω)	R_{ct} (Ω)	CPE-T (S•s^CPE-P)	CPE-P
Ni-Co@Fe-Co PBA	3.86	9.48	7.37E-07	0.80
NiFe-Co PBA	4.23	10.69	4.08E-07	0.86
Ni-Co PBA	3.88	13.88	5.65E-07	0.85
Fe-Co PBA	4.95	16.14	5.77E-07	0.82

Table S8. The value and error of each component in the equivalent circuit for Fe-Co PBA, Ni-Co PBA, NiFe-Co PBA and Ni-Co@Fe-Co PBA in a three-electrode configuration in alkaline simulated seawater (1 M KOH + 0.5 M NaCl).

	R_s (Ω)	R_{ct} (Ω)	CPE-T (S•s^CPE-P)	CPE-P
Ni-Co@Fe-Co PBA	3.36	10.08	3.26E-07	0.86
NiFe-Co PBA	3.41	15.03	2.28E-07	0.88
Ni-Co PBA	3.72	15.57	3.55E-07	0.85
Fe-Co PBA	5.23	32.30	5.18E-07	0.82

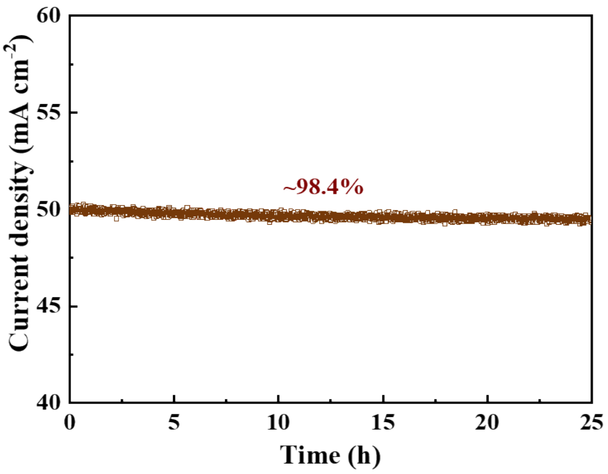

Figure S7. Chronoamperometry measurement of Ni-Co@Fe-Co PBA at an applied potential of -0.23 V vs. RHE in alkaline simulated seawater (1 M KOH + 0.5 M NaCl).

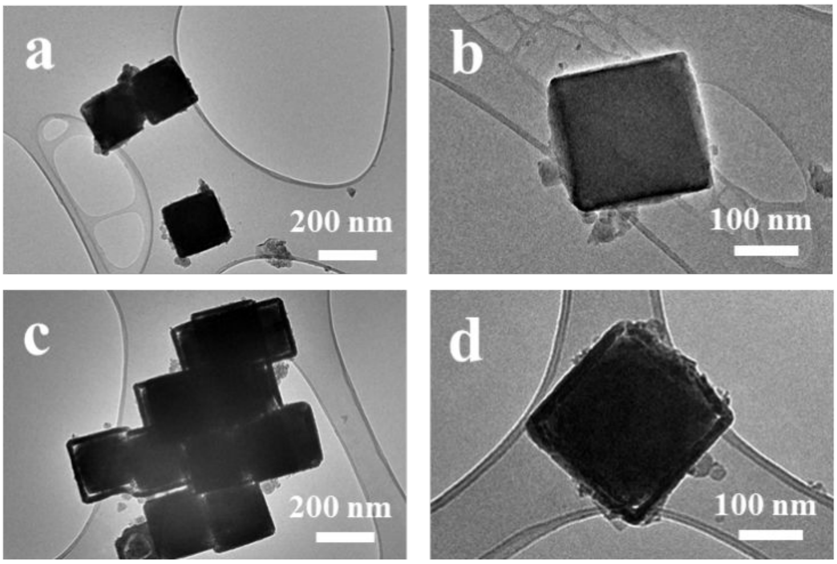

Figure S8. TEM images of (a, b) NiFe-Co PBA and (c, d) Ni-Co@Fe-Co PBA after the long-term stability test at -0.1 V vs. RHE in alkaline freshwater (1 M KOH).

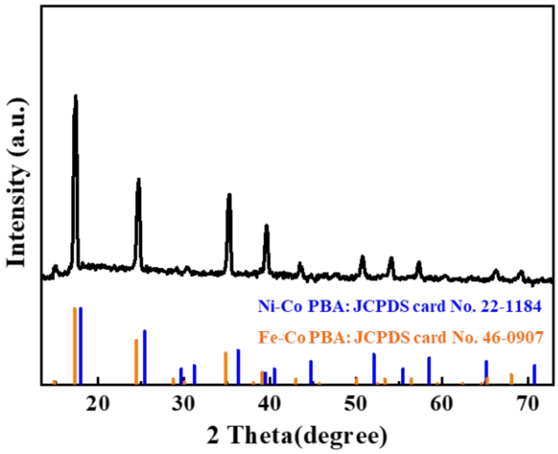

Figure S9. XRD pattern of Ni-Co@Fe-Co PBA after long-term stability test in alkaline simulated seawater (1 M KOH + 0.5 M NaCl).

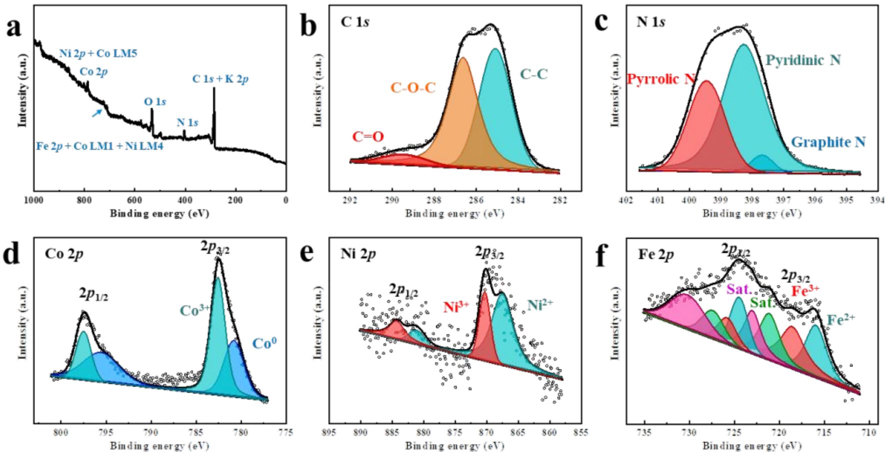

Figure S10. XPS survey scan and high-resolution spectra at (b) C 1*s* edge, (c) N 1*s* edge, (d) Co 2*p* edge, (e) Ni 2*p* edge and (f) Fe 2*p* edge of Ni-Co@Fe-Co PBA after long-term stability test in alkaline simulated seawater (1 M KOH + 0.5 M NaCl).

Table S9. The peak positions and relative contents of chemical bonds and valence of deconvoluted C 1*s*, N 1*s*, Co 2*p*, Ni 2*p* and Fe 2*p* edges for post-HER Ni-Co@Fe-Co PBA.

Catalysts		Post-HER Ni-Co@Fe-Co PBA	
		B.E. (eV)	Area (%)
C 1*s*	C=O	289.6	7
	C-O-C	286.6	44
	C-C	285.1	49
N 1*s*	Pyrrolic N	399.3	38
	Pyridinic N	398.3	56
	Graphite N	397.7	6
Co $2p_{1/2}$	Co^{3+}	797.5	13
	Co^{0}	795.7	21
Co $2p_{3/2}$	Co^{3+}	782.6	36
	Co^{0}	780.8	30
Ni $2p_{1/2}$	Ni^{3+}	884.4	9
	Ni^{2+}	881.4	8
Ni $2p_{3/2}$	Ni^{3+}	870.4	36
	Ni^{2+}	867.6	47
Fe $2p_{1/2}$	Fe^{3+}	725.9	20
	Fe^{2+}	724.5	24
Fe $2p_{3/2}$	Fe^{3+}	718.7	29
	Fe^{2+}	716.0	27

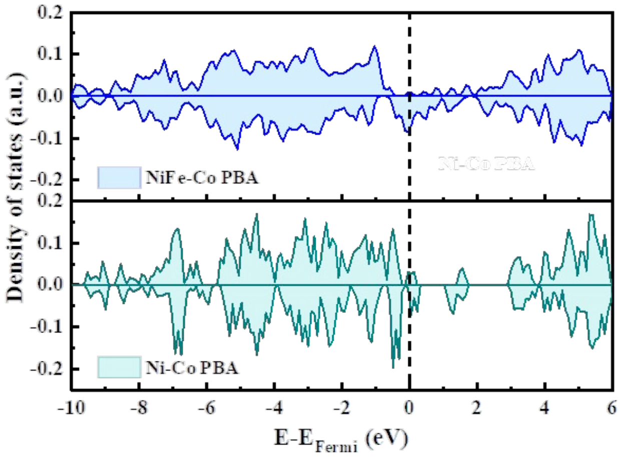

Figure S11. Total density of states (TDOS) of NiFe-Co PBA and Ni-Co PBA.

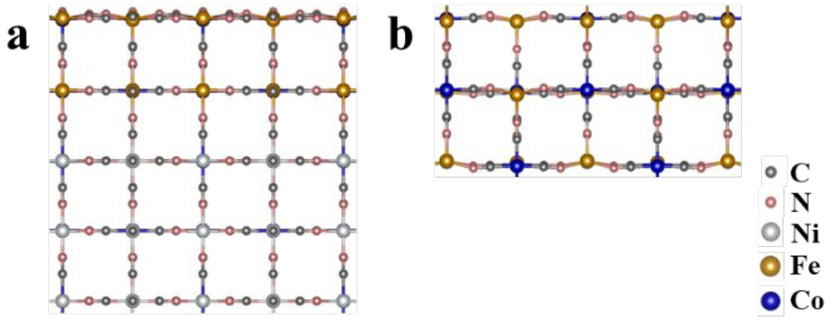

Figure S12. The side and top views of the modeled Ni-Co@Fe-Co PBA structure.

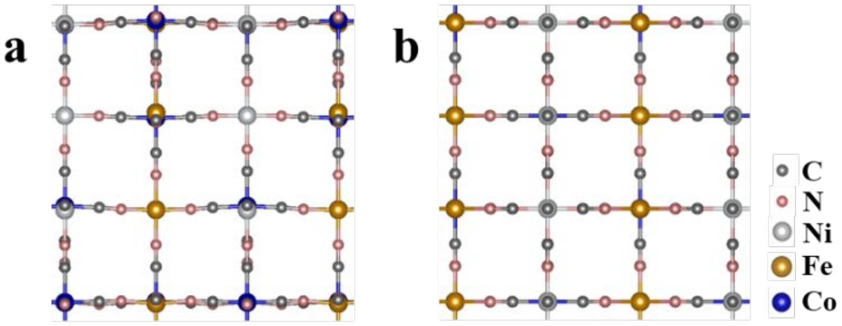

Figure S13. The side and top views of the modeled NiFe-Co PBA structure.

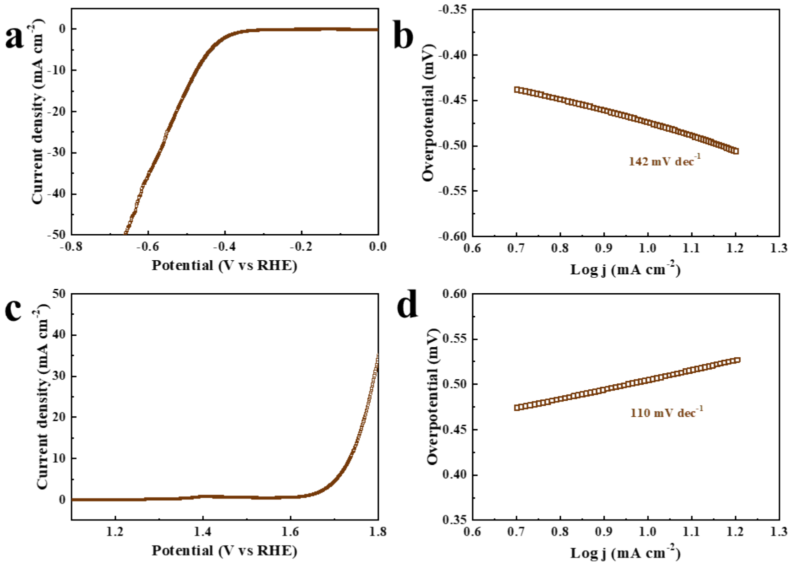

Figure S14. (a) HER polarization curve, (b) HER Tafel plot, (c) OER polarization curve and (d) OER Tafel plot of Ni foam in alkaline freshwater (1 M KOH).

The η_{10} overpotentials and Tafel slopes of nickel foam for HER and OER in alkaline freshwater were 476 mV and 142 mV dec^{-1}, and 507 mV and 110 mV dec^{-1}, respectively, which were much higher than the those of the PBA catalysts, and thus the contribution of nickel foam to water splitting activity was very limited.

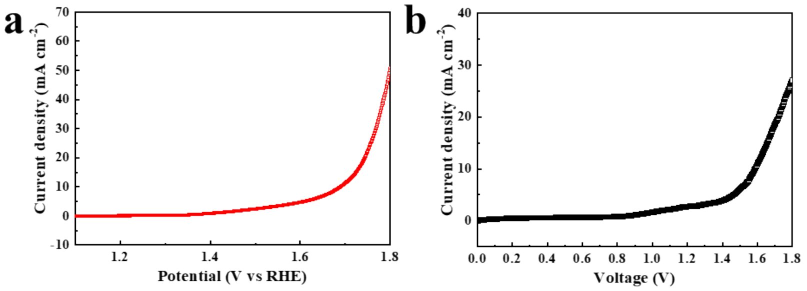

Figure S15. (a) OER polarization curve of Ni-Co@Fe-Co PBA in alkaline freshwater (1 M KOH). (b) Overall water-splitting performance of Ni-Co@Fe-Co PBA//Ni-Co@Fe-Co PBA electrode couple in alkaline freshwater (1 M KOH).

The η_{10} overpotential of Ni-Co@Fe-Co PBA for OER was 428 mV in alkaline freshwater (Figure S15a). The Ni-Co@Fe-Co PBA//Ni-Co@Fe-Co PBA electrode couple reached a current density of 10 mA cm^{-2} at a cell voltage of 1.6 V in alkaline freshwater (Figure S15b), which was significantly inferior to that of the Ni-Co@Fe-Co PBA//NiCo@NiCo-PBA-AA electrode couple.

Table S10. HER activity comparison among Ni-Co@Fe-Co PBA catalyst and other reported PBA-derived electrocatalysts in alkaline freshwater (1 M KOH).

Precursors	Catalysts	Electrolyte	η_{10} (mV vs RHE)	Tafel slope (mV dec^{-1})	Ref.
**Ni-Co PBA**	**Ni-Co@Fe-Co PBA**	**1 M KOH**	**43**	**53**	**this work**
**Ni-Co PBA**	**Ni-Co@Fe-Co PBA**	**1 M KOH + 0.5 M NaCl**	**183**	**60**	
Ni-Co PBA	NiCo@A-NiCo-PBA-AA	1 M NaOH	173	74	*Adv. Funct. Mater.* **2021**, *31*, 2106835
Ni-Co PBA	Pd-e-NiCo-PBA-C	1 M KOH	147	67	*Adv. Funct. Mater.* **2021**, *31*, 2008989
Ni-CoFe PBA	(NiCoFe)CF-MOF	1 M KOH	270	114	*Adv. Funct. Mater.* **2018**, *28*, 1802129
Ni-Fe PBA	NG-NiFe@MoC_2	1 M KOH	150	88	*Nano Energy* **2018**, *50*, 212
Fe-Co PBA	Fe-CoP HTPAs	1 M KOH	230	69	*Small* **2018**, *14*, 1704233
Ni-Fe PBA	3D PBA phosphide	1 M KOH	121	67	*Adv. Energy Mater.* **2018**, *8*, 1800484
Co-Fe PBA	Co-Fe PBA NS	1 M KOH	67	66	*Carbon* **2019**, *142*, 196
Co-Fe PBA	Co^{2+}-rich CoFe-oxide	1 M KOH	284	76	*Appl. Catal. B* **2019**, *258*, 117968
Ni-Co PBA	Fe–Co–Ni–P-1	1 M KOH	215	72	*Inorg. Chem.* **2021**, *60*, 11661

Table S11. Water splitting activity comparison among Ni-Co@Fe-Co PBA//NiCo@A-NiCo-PBA-AA electrode couple and other reported PBA-derived electrocatalysts in alkaline freshwater (1 M KOH).

Precursors	Catalysts	Elelctrolyte	Voltage (V)	Current density (mA cm^{-2})	Ref.
**Ni-Co PBA**	**Ni-Co@Fe-Co PBA//NiCo@A-NiCo-PBA-AA**	**1 M KOH**	**1.6**	**44**	**this work**
	**Ni-Co@Fe-Co PBA//NiCo@A-NiCo-PBA-AA**	**1 M KOH + 0.5 M NaCl**	**1.6**	**30**	
Ni-Co PBA	NiCo@A-NiCo-PBA-AA//NiCo@A-NiCo-PBA-AA	1 M KOH	1.6	13.7	*Adv. Funct. Mater.* **2021**, *31*, 2106835
Ni-Co PBA	Pd-e-NiCo-PBA-C//Pd-e-NiCo-PBA-C	1 M NaOH	1.6	33	*Adv. Funct. Mater.* **2021**, *31*, 2008989
Co-Fe PBA	Co_3S_4@MoS_2//Co_3S_4@MoS_2	1 M KOH	1.6	~12.5	*Nano Energy* **2018**, *47*, 494
Co-Fe PBA	PBA@$Co(OH)_2$//PBA@$Co(OH)_2$	1 M KOH	1.6	~11.2	*Adv. Energy Mater.* **2019**, *9*, 1802939
Ni-Co PBA/Fe-Co PBA	FeCoNi alloys//FeCoNi alloys	1 M KOH	1.6	~9.6	*ACS Catal.* **2017**, *7*, 469
CoFe-PBA	Fe-doped CoP//Fe-doped CoP	1 M KOH	1.6	~10.8	*Small* **2018**, *14*, 1704233
Co-Fe PBA	CoFeP//CoFeP	1 M KOH	1.6	~13	*Chem. Sci.* **2019**, *10*, 464
Ni-Co PBA	Co-Ni-B-O//Co-Ni-B-O	1 M KOH	1.6	~6.3	*J. Mater. Chem. A* **2018**, *6*, 23289

Table S12. HER activity comparison among Ni-Co@Fe-Co PBA catalyst and other recently reported non-noble-metal-based electrocatalysts in alkaline (simulated or natural) seawater.

Catalysts	Electrolyte	η_{10} (mV vs RHE)	η_{100} (mV vs RHE)	Tafel slope (mV dec^{-1})	Ref.
**Ni-Co@Fe-Co PBA**	**1 M KOH + 0.5 M NaCl**	**183**	**258**	**60**	**this work**
NCMS/NiO	1 M KOH + Seawater	/	~280	/	*J. Mater. Chem. A* **2022**, *10*, 9547
NiFeP-NS	1.0 M KOH + 1.0 M NaCl +0.01 M $NaHCO_3$	/	126	97	*Appl. Catal., B* **2022**, *302*, 120862
Fe,P-$NiSe_2$ NFs	Seawater	/	~640	/	*Adv. Mater.* **2021**, *33*, 2101425
Ni-SN@C	1 M KOH + Seawater	23	~180	41	*Adv. Mater.* **2021**, *33*, 2007508
Ni-SA/NC	1 M KOH + Seawater	139	~310	123	*Adv. Mater.* **2021**, *33*, 2003846
NiMoN	1 M KOH + Seawater	/	82	/	*Nature Commun.* **2019**, *10*, 5106
NiFe-PBA-gel-cal	1 M KOH + 0.5 M NaCl	/	480	161	*Adv. Sci.* **2022**, *9*, 2200146
Mo_5N_6	Seawater	258	/	/	*ACS Nano* **2018**, *12*, 12761

Table S13. Water splitting activity comparison among Ni-Co@Fe-Co PBA//NiCo@A-NiCo-PBA-AA electrode couple and other recently reported two-electrode systems in alkaline (simulated or natural) seawater.

Catalysts	Electrolyte	U_{10} (mV vs RHE)	U_{100} (mV vs RHE)	Ref.
**Ni-Co@Fe-Co PBA//NiCo@A-NiCo-PBA-AA**	**1 M KOH + 0.5 M NaCl**	**191**	**477**	**this work**
NCMS/NiO//NCMS/NiO	1 M KOH + Seawater	/	527	*J. Mater. Chem. A* **2022**, *10*, 9547
NiFeP-NW//NiFeP-NS	1.0 M KOH + 1.0 M NaCl +0.01 M $NaHCO_3$	/	~420	*Appl. Catal., B* **2022**, *302*, 120862
Fe,P-$NiSe_2$ NFs//Fe,P-$NiSe_2$ NFs	Seawater	/	369	*Adv. Mater.* **2021**, *33*, 2101425
Ni-SN@C//Ni-SN@C	1 M KOH + Seawater + 0.1 M N_2H_4	366	/	*Adv. Mater.* **2021**, *33*, 2007508
NiMoN@NiFeN//NiMoN	1 M KOH + Seawater	/	351	*Nature Commun.* **2019**, *10*, 5106
NiFe-PBA-gel-cal//NiFe-PBA-gel-cal	1 M KOH + 0.5 M NaCl	/	430	*Adv. Sci.* **2022**, *9*, 2200146

References

1. Zhang, H.; Li, P.; Chen, S.; Xie F.; Riley, D. J. Anodic Transformation of a Core-Shell Prussian Blue Analogue to a Bifunctional Electrocatalyst for Water Splitting. *Adv. Funct. Mater.* **2021**, *31*, 2106835.
